# Construction of a high-density genetic map and QTLs mapping for sugars and acids in grape berries

**DOI:** 10.1186/s12870-015-0428-2

**Published:** 2015-02-03

**Authors:** Jie Chen, Nian Wang, Lin-Chuan Fang, Zhen-Chang Liang, Shao-Hua Li, Ben-Hong Wu

**Affiliations:** Beijing Key Laboratory of Grape Science and Enology, and CAS Key Laboratory of Plant Resources, Institute of Botany, Chinese Academy of Sciences, Beijing, 100093 P. R. China; University of Chinese Academy of Sciences, Beijing, 100049 P. R. China; Key Laboratory of Plant Germplasm Enhancement and Speciality Agriculture, Wuhan Botanical Garden, Chinese Academy of Sciences, Wuhan, 430074 China

**Keywords:** Berry quality, Genetic map, Next-generation sequencing (NGS), QTL analysis, Quantitative trait loci, Restriction-site associated DNA (RAD), *Vitis*

## Abstract

**Background:**

QTLs controlling individual sugars and acids (fructose, glucose, malic acid and tartaric acid) in grape berries have not yet been identified. The present study aimed to construct a high-density, high-quality genetic map of a winemaking grape cross with a complex parentage (*V. vinifera* × *V. amurensis*) × ((*V. labrusca* × *V. riparia*) × *V. vinifera*), using next-generation restriction site-associated DNA sequencing, and then to identify loci related to phenotypic variability over three years.

**Results:**

In total, 1 826 SNP-based markers were developed. Of these, 621 markers were assembled into 19 linkage groups (LGs) for the maternal map, 696 for the paternal map, and 1 254 for the integrated map. Markers showed good linear agreement on most chromosomes between our genetic maps and the previously published *V. vinifera* reference sequence. However marker order was different in some chromosome regions, indicating both conservation and variation within the genome. Despite the identification of a range of QTLs controlling the traits of interest, these QTLs explained a relatively small percentage of the observed phenotypic variance. Although they exhibited a large degree of instability from year to year, QTLs were identified for all traits but tartaric acid and titratable acidity in the three years of the study; however only the QTLs for malic acid and β ratio (tartaric acid-to-malic acid ratio) were stable in two years. QTLs related to sugars were located within ten LGs (01, 02, 03, 04, 07, 09, 11, 14, 17, 18), and those related to acids within three LGs (06, 13, 18). Overlapping QTLs in LG14 were observed for fructose, glucose and total sugar. Malic acid, total acid and β ratio each had several QTLs in LG18, and malic acid also had a QTL in LG06. A set of 10 genes underlying these QTLs may be involved in determining the malic acid content of berries.

**Conclusion:**

The genetic map constructed in this study is potentially a high-density, high-quality map, which could be used for QTL detection, genome comparison, and sequence assembly. It may also serve to broaden our understanding of the grape genome.

**Electronic supplementary material:**

The online version of this article (doi:10.1186/s12870-015-0428-2) contains supplementary material, which is available to authorized users.

## Background

The organoleptic quality of table grapes and the flavor and stability of wine depend strongly on the types of sugars and acids, as well as the total sugar and acid concentration, in the grapes. Generally, fructose and glucose are predominant in berries at maturity, and sucrose is present in smaller quantities [1-3]. They have different levels of sweetness: if sucrose is rated 1, then fructose is 1.75 and glucose 0.75 [4-6]. The main organic acids in grape berries are tartaric and malic acids, which typically account for 90% of total acids [7-9]. Malic acid is involved in many processes that are essential for the health and sustainability of the vine, and tartaric acid plays an important role in maintaining the chemical stability and the color of the wine. Tartaric acid has a stronger acidic flavor than malic (pKa: 3.04 vs. 3.40), and is also more sour [10].

Many studies have identified genomic loci that are linked to traits of interest in grapes. Modern strategies for the investigation of loci are based on the construction of genetic linkage maps, which was facilitated by the development of molecular markers. The first maps were constructed based mainly on RAPD [11] and AFLP [12] markers. Since then, a range of markers has been developed, and genetic maps of various grape cultivars and other *Vitis* species have been constructed [13-32]. One of these, a genetic map of a *V. vinifera* cross between Syrah and Pinot Noir, took into account most markers, including 483 SNP, 132 SSR and 379 AFLP markers [31]. Wang *et al.* [33] developed a genetic map with a total of 1 814 SNP markers. For a single SNP marker, the lowest integrity was ~85%. Of these 1 814 SNP markers, 1 545 were homozygous for one parent and heterozygous for the other (960 for lm×ll and 585 for nn×np), constituting 85.2% of all selected SNP markers. However, the other three types of markers that could be mapped on both female and male linkage maps amounted to 14.8% (ab×cd: 77, ef×eg: 171 and hk×hk: 21) [33]. Of these, 1 121 are on the female map, 759 are on the male map, and 1 646 are on the integrated map. This map was produced by combining next generation sequencing (NGS) and restriction-site associated DNA (RAD). Recently, Barba *et al.* [34] also used NGS to construct linkage maps for *V. rupestris* B38 and ‘Chardonnay’, with 1 146 and 1 215 SNPs each, covering 1 645 and 1 967 cM, respectively, and asserting that NGS was a powerful method for constructing a high-density, high-quality genetic map.

In grapes, quantitative trait loci (QTL) detection has mostly been used to investigate the genes related to resistance to diseases such as powdery and downy mildew and Pierce’s disease [20,26,29,35-37], as well as pest resistance [19,20,38-41]. It has also been used to examine the genes related to a range of agronomic traits, e.g. berry size, seed number, mean and total seed fresh and dry weights, berry weight [14,17,20,27,39,42,43], inflorescence and flower morphology, number of inflorescences per shoot, flowering date [26], timing and duration of flowering and of veraison, veraison-ripening interval [14,44], architecture of the inflorescence [45], aroma profile [46], anthocyanin content [47], and number of clusters per vine [42]. In addition, the QTLs controlling sexual traits [26] and fertility [48] have been identified.

The genes controlling sugar and acid production in grapes are extremely complex, because of both the diverse chains of metabolic processes involved and the effect of environmental factors influencing these processes [49]. Viana *et al.* [50] have recently identified some QTLs involved in controlling soluble solid concentrations, pH, and titratable acidity in grape berries, but these explain a small amount of phenotypic variation in these traits. To our knowledge, no QTLs controlling the production of individual sugars and acids in grape berries have yet been identified. Some analyses of QTLs controlling soluble solid concentrations, titratable acidity, pH and the production of individual sugars and acids have, however, been conducted for other fruit tree species, such as peach [51,52], apple [53-56], sour cherry [57] and melon [58].

The aim of this work was to investigate the genetic determination of soluble solid concentrations, titratable acidity, and individual sugars and acids in grape berries. A high-density genetic map was constructed for the population, as described in Wang *et al.* [33]. The map was used in combination with phenotypic data to identify marker-linked loci, after which we identified loci related to phenotypic variability observed over three years. This population was derived from the interspecies cross of cultivars ‘Beihong’ (BH) and ‘E.S.7-11-49’ (ES).

## Methods

### Plant material

The population, which comprised 1 200 individuals, was obtained by crossing BH (*Vitis vinifera* ‘Muscat Hamburg’ × *V. amurensis*) with ES ((Minnesota 78 (*V. labrusca* ‘Beta’ × Witt) × *V. riparia*) × *V. vinifera* ‘Chenin Blanc’) in 2007. We randomly selected 249 individuals for our experiment, and used these to construct the genetic map. Due to plant mortality, poor fruit setting, and environmental factors (e.g. rainfall, hail storms), the number of individuals bearing fruits varied from year to year. Vines were planted in 2008, without replicate, in the vineyard at the Institute of Botany, Chinese Academy of Sciences, Beijing (39°90' N 116°30' E). They were trained to fan-shaped trellises and had single trunks, which facilitated protection during winter. The vines were spaced 1.0 m apart within the row and 2.5 m apart between rows, and rows were north–south oriented. They were maintained under routine cultivation conditions, including irrigation, fertilization, soil management, pruning and disease control.

A random set of fruiting genotypes and the two parents were used in each of the three years of the study (2011–2013). In total, 241 genotypes were used in 2011, 225 in 2012, and 197 in 2013 for phenotypic measurement. Of these, 187 were common to all three years. Three replicates of one or two berry clusters were harvested from each genotype and parent at maturity. Maturity date was estimated primarily by assessing the physical properties of the berries, the ease of removal of berries from pedicels (without berry tissue shriveling because of loss of water), and the change of seed color from bright green to tan-brown [59]. Date of maturity was also estimated partly based on previous records. In addition, by the same person was responsible for berry harvesting for the duration of the study, to ensure consistency in the estimation of date of maturity. Maturity date ranged from 15 August to 15 September in 2011, and from 20 August to 20 September in both 2012 and 2013, depending on the genotype. Harvested clusters were placed in plastic bags on ice and transported immediately to the laboratory, which took ~10 min. This mode of transportation did not result in significant change in tartaric acid concentration relative to normal transportation.

### Measurement of sugars and acids

Each replicate was pressed using a hand juicer to extract berry juice. Soluble solids concentration (SSC, °Brix) of the juice was measured with a digital hand-held refractometer (Atago, Tokyo, Japan). A 2 mL sample of juice was diluted to 10 mL with deionized water, and titratable acidity was measured by titration up to pH 8.2 with 0.1 mol·L^−1^ NaOH, and expressed as g·L^−1^ of tartaric acid.

The remaining juice was centrifuged at 5 000 g for 15 min. The supernatants were decanted, passed through a SEP-C18 cartridge (Superclean ENVI C18 SPE), and filtered through a 0.22 μm Sep-Pak filter. The sugar and acid concentrations of the filtered supernatants were measured using a Dionex P680 HPLC system (Dionex Corporation, CA, USA).

Fructose and glucose concentrations were measured using a Shodex RI-101 refractive index detector with a Waters Sugar-Pak I column (300 mm × 6.5 mmI.D., 10 μm particle size) and a guard column cartridge (Sugar-Pak I Guard-Pak Insert, 10 μm particle size). The reference cell was maintained at 40°C. The column was maintained at 90°C using a Dionex TCC-100 thermostated column compartment. Degassed, distilled, deionized water at a flow rate of 0.6 mL·min^−1^ was used as the mobile phase. The injection volume was 10 μL.

Malic and tartaric acid concentrations were measured using a Dionex UltiMate3000 detector, with a Dikma PLATISIL ODS column (250 mm × 4.6 mmI.D., 5 μm particle size) and a guard column cartridge (DikmaSpursil C18 Guard Cartridge 3μm, 10 mm × 2.1 mm). The column was maintained at 40°C. Samples were eluted with 0.02 mol·L^−1^ KH_2_PO_4_ solution with pH 2.4, at a flow rate of 0.8 mL·min^−1^. Eluted compounds were detected using UV absorbance at 210 nm.

The Chromeleon chromatography data system was used to integrate peak areas according to external standard solution calibrations [60] (reagents from Sigma Chemical Co. Castle Hill, NSW, Australia). Sugar and acid concentrations were expressed in mg·mL^−1^ juice.

### DNA extraction

Young leaves (the second and third leaf from the apex) were harvested from each genotype and the two parents at the beginning of the vegetative period (late spring). The samples were immediately stored in liquid nitrogen and transferred to a freezer maintained at −80°C. Samples, weighing 0.5 g were ground in liquid nitrogen and genomic DNA was extracted using DNeasy plant mini prep kit (Qiagen). Briefly, 2 μg genomic DNA from each sample (249 F1 progeny and both parents) was treated with 20 units (U) MseI (New England Biolabs [NEB]) for 60 min at 37°C in a 50 μL reaction. A quick blunting kit (NEB) was used to convert 30 μL of the digested sample to 5’-phosphorylated, blunt-ended DNA in a 50 μL reaction mixture; the reaction was performed with 30 μL of digested sample, 5 μL 10× blunting buffer, 5 μL 1 mM dNTP mix, 2 μL blunting enzyme mix and 8 μL sterile dH_2_O at room temperature for 30 min. A 3’-adenine overhang was added to the resulting samples in a 50 μL reaction with 32 μL blunt-ended DNA sample, 5 μL Klenow buffer (10×), 10 μL dATP (1 mM), 3 μL Klenow fragments (3’→5’exo-, 5 U·μL^−1^) and sterile dH_2_O to the final volume at 37°C for 1 h. Then 2 μL of 100 nM P1 and P2 adapter with a 3- to 5- bp plant-specific index (barcode) at the 5’ end and a thymine overhang at the 3’ end was added to each sample in a 50 μL reaction. A ligation reaction was carried out overnight at 16°C with T4 DNA ligase and 16 samples with different plant indices pooled into one. DNA fragments of 400–500 bp (including the ~120 bp adaptor) were separated on a 1.5% agarose gel and purified using a MiniElute gel extraction kit (Qiagen). Finally, all pooled samples were amplified with Phusion High-Fidelity PCR Master Mix (NEB) for 18 cycles in a 100 μL reaction including 20 μL Phusion master mix, 5 μL of 10 μM modified Solexa amplification primer mix (AP1 and AP1; 2006 Illumina, Inc., allright reserved) and sterile dH_2_O to the final volume. The AP1 and AP2 primers contained Illumina paired end sequencing primer sites. DNA concentration was measured using a 2.0 fluorometer at BGI (Beijing Genomics Institute, China) [33].

### High-throughput genotyping and map construction

High-density genetic maps for the two parents, BH and ES, were constructed using a slightly altered version of the method described by Wang *et al.* [33]. All experiments were performed at BGI. RAD-seq libraries for all 249 genotypes and the two parents were constructed according to Etter *et al.* (2011) [61], and sequenced using the Illumina HiSeq 2000 platform. The raw data produced were filtered to remove adaptors, indices and low-quality data (reads with > 15% of bases with quality score < 30). The cleaned data were analyzed using a standard RAD-seq analysis pipeline in the software package Stacks [62]. Genotypes for each plant in the population were assigned according to these results. Representative sequences for each SNP marker were obtained based on sequence clustering during the RAD-seq analysis pipeline. To manage the large quantity of data, a number of custom-programmed Perl scripts were also used to conduct the analysis.

To identify anchor markers for this study, we first identified a set of SNP markers, which we used to assign the 19 grapevine chromosomes to 19 linkage groups (LGs). This was done in two steps. Firstly, we marked the segregation patterns of all identified SNP markers as ab × cd, ef × eg, hk × hk, lm × ll, and nn × np. The first three of these pairs, which appeared in both parental linkage maps, were treated as candidate anchor markers. Secondly, because all alleles of each SNP marker had two nearly identical 100 bp sequences, the sequences from any allele could be taken as representative of the genotype of this SNP marker. These two representative sequences from the candidate anchor markers were aligned with the sequence of the 12× genomic assembly for *V. vinifera* PN40024, using local BLAST software with parameters set to –m 8 and –e 1E-5. The positions of each sequence for one SNP marker on the genome were identified based on their top hit. Three strict criteria were used to select anchor markers: 1) the marker had to show no significant segregation distortion among the 249 progeny genotypes in our population (*P* < 0.001); 2) both of the marker’s end sequences had to align with the same chromosome position on the physical map for the reference PN40024 genome; and 3) the distance between the positions for the two end sequences on the reference genome had to fall between 200 and 500 bp (the expected size of the digested fragments was ~300–400 bp).

In constructing the map, the double pseudo-test cross strategy of Grattapaglia and Sederoff [63] was applied, using JoinMap4.0 (Kyazma). After data had been imported, a cross pollination (CP) model was used for data mining. The ratio of marker segregation was calculated using Chi-squared tests. Firstly, markers that showed significantly distorted segregation (*P* < 0.001) were excluded from further analyses; secondly, marker order on each linkage group was optimized by excluding markers with *χ*^2^ > 3.0. The genotypes of 1 826 SNP markers were analyzed for linkage and recombination, using the Kosambi function to estimate genetic map distances. Logarithm of odds (LOD) score thresholds ≥ 7 was used to group the markers. After the LGs had been computed, their number was assigned according to the anchor markers mapped on them.

### QTL analysis

All trait data were Box-Cox transformed to unskew their distributions, and the normality of the distributions was tested using the Shapiro-Wilks test. The detection of QTLs using both the transformed and the original data yielded similar results in terms of number, location and contribution of QTLs, so the original data were henceforward used and reported.

QTLs for all traits in the population in the three separate years were analyzed for the parents only using the composite interval mapping (CIM) method in WinQTL Cartographer 2.5 [64,65]. CIM was used to scan the genetic map and estimate the likelihood of a QTL and its corresponding effect for every 1 cM. The forward regression algorithm was used to identify cofactors. A thousand permutations were performed using the CIM model within, and the thresholds for each environment were identified (almost all environments had thresholds at LOD ~3.0; *P* ≤ 0.05). The 1-LOD confidence interval within the CIM model corresponded to the 95% confidence interval calculated by WinQTL Cartographer 2.5 for each QTL. The results showed that when LOD values were 3–3.2, the error rate was 5%. Threshold LOD value was therefore set to 3 for all traits. QTLs with peaks close to 5 cM were merged into one QTL, and each significant QTL was characterized by its maximum LOD score, the percentage of variation it explained and its confidence intervals in cM, corresponding to the maximum LOD score withinone unit’s width either side of the LOD peak.

### Search for candidate genes

For each QTL, the search for candidate genes was conducted in the genomic region corresponding to the confidence interval determined on the consensus map. The scrutinized sequence was limited by the most proximal SNP markers that were present in both the reference genome and the consensus map. The genes were selected based on the information available for the annotated reference genome (Genoscope 12×) of the quasi-homozygous line 40024 derived from Pinot noir (http://www.genoscope.cns.fr/externe/GenomeBrowser/Vitis/) [66]. They were classified according to their biological function as registered in the database. The genes catalogued as “unknown function” or equivalent were not considered in further analyses. In addition, a gene ontology (GO) enrichment analysis was performed, considering the genes identified in the physical genomic region that was associated with the confidence interval for each QTL. We also compared the frequency of each QTL vs. the complete reference genome, and searched for possible enrichment in gene functions. All enrichment analyses were done with the agriGO tool (http://bioinfo.cau.edu.cn/agriGO), using the options “singular enrichment analysis” and “complete GO”. Significant GO terms (*P* < 0.05) were calculated using a hypergeometric distribution and the Yekutieli multi-test adjustment method [67].

### Statistical analysis

Glucose-to-fructose ratio and β ratio (tartaric acid-to-malic acid ratio) were calculated, as these have been proposed as useful descriptors for evaluating the sugar and acid composition of grape berries [3,68]. For all further analyses, the means of the three replicates for each genotype and the parents were used.

All statistical analyses were performed using S-Plus (MathSoft Inc.). The frequency distribution of each trait was analyzed using the function ‘hist’, and the number of classes was determined using the Sturges method. Phenotypic correlations between traits within years and between years for each trait were calculated using the non-parametric Spearman correlation coefficient.

## Results

### Phenotypic characterization of parents and individuals

Averaged correlation coefficients between each pair of years were significant at *P* < 0.001 for almost all traits, ranging from 0.52 for the glucose-to-fructose ratio, to 0.74 for titratable acidity (Table 1).

**Table 1:** **Phenotypic correlation coefficients between the traits of grape berries produced by crossing ‘Beihong’ with ‘E.S.7-11-49’**

	**Fructose**	**Glucose**	**Total sugar**	**SSC**	**G/F**	**Tartaric**	**Malic**	**Total acid**	**TA**	**β ratio**
Fructose	0.56***	0.93***	0.98***	0.86***	−0.27(ns)	−0.32(ns)	−0.52***	−0.49***	−0.57***	0.25(ns)
Glucose		0.57***	0.98***	0.86***	ns(+)	−0.32 (ns)	−0.48***	−0.47***	−0.55***	0.20 (ns)
Total sugar			0.56***	0.87***	ns	−0.32 (ns)	−0.52***	−0.49***	−0.57***	0.23 (ns)
SSC				0.63***	ns	−0.32***	−0.54***	−0.54***	−0.56***	0.20 (ns)
G/F					0.53***	ns	ns(+)	ns	ns	ns(−)
Tartaric						0.63***	0.36***	0.76***	0.59***	0.39(ns)
Malic							0.71***	0.88***	0.82***	−0.56***
Total acid								0.68***	0.88***	−0.27(ns)
TA									0.74***	−0.30***
β ratio										0.64***

Correlation coefficients were averaged over three years, and over 241 genotypes in 2011, 225 in 2012, and 197 in 2013 (except for TA in 2013, for which there were 189 genotypes). The averages of the correlation coefficients between each two-year combination (2011 and 2012, 2011 and 2013, 2012 and 2013) for each trait are shown in the diagonal. SSC is the soluble solids content, G/F is the glucose-to-fructose ratio, TA is titratable acidity, and β ratio is the tartaric acid-to-malic acid ratio.

***Significant at *P*<0.001 in all three years.

ns: not significant and/or significant at *P*<0.05 in all three years.

ns (+/−): significant (+ = positive, − = negative) only in one year at *P*< 0.001 or *P*< 0.01.

ns (+) in diagonal: significant only between 2011 and 2012.

(ns): not significant only in one year; the correlation coefficients significant at *P*< 0.001 or *P*< 0.01 for the other two years were averaged.

Fructose, glucose, total sugar and SSC were positively correlated with each other. Fructose and glucose were strongly positively correlated, with a correlation coefficient of 0.93 (*P* < 0.001). The glucose-to-fructose ratio, however, was inconsistently correlated with fructose and glucose over the three years, and was not significantly correlated with total sugar, SSC or the acid-related traits. There were significant positive correlations between tartaric acid, malic acid, total acid and titratable acidity, from 0.36 between tartaric acid and malic acid to 0.88 between total acid and titratable acidity. The β ratio was significantly negatively correlated with malic acid and titratable acidity, but did not have consistent relationships with tartaric or total acid. The sugar-related and acid-related traits were, in general, negatively correlated, but the sugar-related traits were weakly positively correlated with the β ratio.

**Figure 1:**
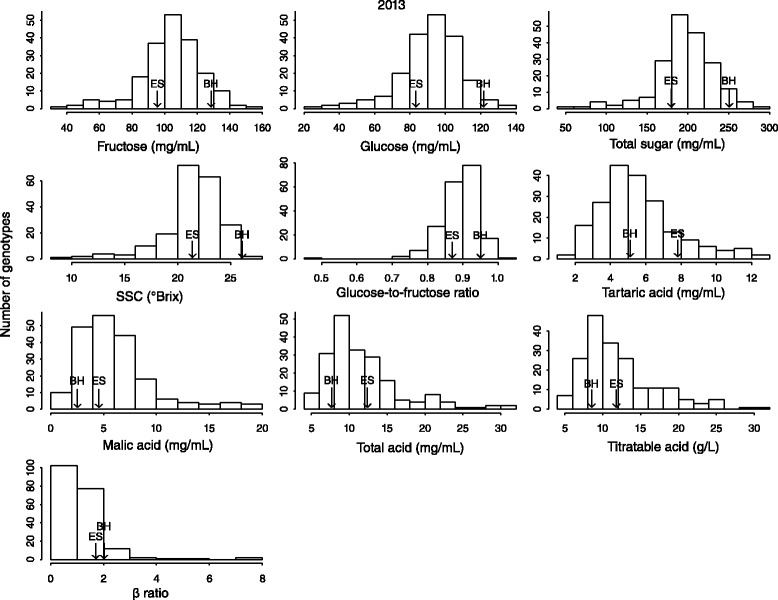
**Distribution of traits of the F1 population derived from the cross ‘Beihong’ (BH) × ‘E.S.7-11-49’ (ES) in 2013.** There were 197 genotypes in 2013 (189 for titratable acidity), using the averages of three replicates per genotype. The values for the maternal parent, BH, and the paternal parent, ES, are indicated by arrows. SSC and β ratio represent soluble solids content and the tartaric acid-to-malic acid ratio, respectively.

The traits examined showed approximately the same phenotypic data distributions for all three years (Figures 1 and Additional file 1: Figure S1). All traits exhibited continuous variation, which is typical of quantitatively inherited traits. Transgressive segregation was apparent in fructose, glucose, total sugar, SSC, glucose-to-fructose ratio and β ratio traits. For these traits, fewer than 12% of the genotypes had higher phenotypic values than the high-value parent (indeed only one genotype exceeded the parents’ phenotypic value in 2011), and fewer than 29% of genotypes had lower phenotypic values than the low-value parent. Transgressive segregation was more apparent in the tartaric acid, malic acid, total acid and titratable acidity traits; for these traits, 37–88% of genotypes exceeded the high-value parent’s phenotypic value, and 25–58% of genotypes were below the low-value parent.

### Construction of genetic maps

A total of 1 826 SNP-based markers were used to construct the genetic maps. The lowest integrity for a single SNP marker was ~83.0%. Of the 1 826 SNP markers, 1 515 were homozygous for one parent and heterozygous for the other (803 for lm × ll and 712 for nn × np), constituting 83.0% of all selected SNP markers. The remaining 17.0% constituted the other three types of markers that could be mapped on both female and male linkage maps (ab × cd: 1, ef × eg: 109 and hk × hk: 201). The minimum number of reads for an SNP marker to be accepted was five per allele; 181 distorted markers were removed. For the BH map, 621 markers were assembled into 19 LGs spanning 1 553.43 cM of map distance, with an average interval length of 2.50 cM. The ES map was based on 696 markers positioned in 19 LGs, and covered 1 381.02 cM, with an average interval length of 1.98 cM (Table 2, Additional file 2: Figure S2, Additional file 3: Table S1). The integrated map of maternal and paternal LGs included 1 254 markers, unevenly distributed between LGs. The total number of markers per LG ranged from 11 (LG16) to 66 (LG18) for the BH map, and from seven (LG05) to 63 (LG07) for the ES map. Each 1 000 kb of DNA sequence occupied an average of ~3.68 cM on the BH map and ~3.27 cM on the ES map. The average interval between two adjacent mapped markers was estimated at ~679 kb (2.50/3.68 × 1 000) for the BH map, and ~606 kb (1.98/3.27 × 1 000) for the ES map.

**Table 2:** **Genetic map and number of common markers between genetic and physical maps for linkage groups**

	**Number of markers**			**Genetic size (cM)**		**Number of common markers**		**Chromosome size (Mb)**
	**BH**	**ES**	**Integrated**	**BH**	**ES**	**BH**	**ES**	
LG01	25	43	66	81.688	78.187	23	41	22.8
LG02	49	30	75	106.868	67.662	37	25	18.6
LG03	16	30	45	96.402	69.187	15	27	19.2
LG04	46	41	83	106.634	104.325	40	39	23.7
LG05	39	7	46	79.219	22.831	13	7	24.8
LG06	30	47	75	79.630	71.633	27	45	21.3
LG07	13	63	73	25.035	98.122	9	52	20.9
LG08	53	41	88	94.558	73.718	41	41	22.3
LG09	24	33	54	86.867	71.318	23	32	23.0
LG10	45	20	60	81.571	79.890	23	10	17.7
LG11	24	40	60	79.699	74.507	23	40	19.3
LG12	34	53	75	66.618	52.297	29	47	22.3
LG13	33	54	81	79.181	82.256	30	41	24.4
LG14	28	38	65	83.639	90.139	26	36	30.1
LG15	29	22	49	87.052	58.515	14	21	20.1
LG16	11	19	27	50.544	56.048	8	18	21.7
LG17	27	34	60	63.685	58.969	26	34	16.5
LG18	66	52	117	92.401	101.306	54	44	29.3
LG19	29	29	54	112.139	70.110	19	25	23.8
Total	621	696	1254	1553.430	1381.020	480	625	421.8

The number of markers on the 19 linkage groups (LGs) of the ‘Beihong’ (BH) and ‘E.S.7-11-49’ (ES) genetic maps, their genetic sizes, and the number of markers common to both the genetic maps and the physical map for the reference PN40024 genome.

### Comparison of genetic and reference sequences

Of the 1 254 markers used in the integrated genetic map, 1 055 were on the physical map for the reference PN40024 genome (Table 2), which suggests our genetic maps cover 84.1% of the reference genome. Of the 621 markers on the BH map, 480 (77.3%) were common to both the genetic and physical maps, and of the 696 markers on the ES map, 625 (89.8%) were shared (Table 2). The physical size of the corresponding chromosomes ranged from 16.5 Mb (LG17) to 30.1 Mb (LG14). In individual LGs, the number of markers common to both the genetic and physical maps ranged from eight (LG16) to 54 (LG18) for BH, and from seven (LG05) to 52 (LG07) for ES. The positions of the common markers on the genetic maps were compared with their physical positions on the reference genome (Additional file 4: Figure S3, Additional file 3: Table S1). Most of the markers showed good linear agreement between the genetic and physical maps, with exceptions found on a few specific chromosomes (e.g. Chr05 and Chr16).

### QTL identification

QTLs were analyzed separately on the parental maps for each of the three years (Table 3, Additional file 2: Figure S2). The CIM procedure detected 19 QTLs on the BH map, on LG02, LG03, LG06, LG09 and LG18, with 1, 1, 2, 1 and 14 QTLs, respectively. The average LOD value of the QTLs was 4.0, ranging from 3.0–8.1. On the ES map, 19 QTLs were detected on LG01, LG04, LG07, LG11, LG13, LG14, LG17 and LG18, with 1, 1, 1, 1, 1, 10, 3 and 1 QTLs, respectively. Here the average LOD value of the QTLs was 3.9, ranging from 3.0–6.1. The genomic threshold for both maps was ~3.0.

**Table 3:** **Summary of QTLs in F1 population derived from the cross ‘Beihong’ (BH) × ‘E.S.7-11-49’ (ES)**

**Trait**	**Year**	**LG**	**Parent**	**Marker name**	**Peak location**	**LOD score**	***R*** ^***2***^ **(%)**	**95% confidence interval left**	**95% confidence interval right**
Fructose	2011	14	ES	186084	17.51	3.52	8.24	16.90	20.90
	2011	14	ES	66968	23.11	4.20	7.63	22.40	24.50
	2011	17	ES	275393	30.71	3.47	6.27	29.90	32.50
	2012	4	ES	200017	85.31	3.11	5.58	81.90	88.90
	2013	11	ES	201825	72.71	4.21	9.71	72.20	73.90
Glucose	2011	14	ES	17727	16.51	3.00	6.18	16.10	18.00
	2011	14	ES	66968	23.11	4.44	8.16	22.40	24.30
	2011	14	ES	149275	46.21	3.31	6.04	42.90	49.30
Total sugar	2011	14	ES	17727	16.51	3.26	6.72	16.20	18.00
	2011	14	ES	66968	23.11	4.52	8.37	22.40	24.30
	2011	14	ES	149275	46.21	3.19	5.85	43.00	49.10
SSC	2011	14	ES	66968	23.11	6.13	11.42	22.80	25.70
	2011	14	ES	167058	29.81	3.58	7.17	28.10	31.90
	2012	18	BH	120619	47.41	3.08	6.03	45.40	47.80
	2013	1	ES	32402	61.01	3.58	6.96	54.60	65.00
Glucose-to-fructose ratio	2011	2	BH	125072	103.91	3.08	6.33	102.90	104.90
	2011	3	BH	182932	28.41	3.92	6.77	17.00	29.90
	2012	9	BH	117666	40.41	3.18	5.71	39.60	44.70
	2013	7	ES	296046	48.41	4.58	10.87	47.60	52.30
	2013	17	ES	250686	29.91	3.01	5.94	29.10	30.80
	2013	17	ES	76880	41.41	4.91	9.92	40.90	41.60
**Malic acid**	2011	6	BH	248487	74.11	3.68	17.31	72.00	76.90
	2011	18	BH	215808	24.31	5.45	9.49	23.90	26.40
	2011	18	BH	120619	47.41	5.07	9.63	45.50	47.60
	2012	18	BH	280521	23.21	3.68	7.13	22.80	26.80
	2012	18	BH	120619	47.41	6.09	11.83	45.60	47.60
	2012	18	BH	15694	53.51	4.78	8.49	53.20	55.40
Total acid	2011	6	BH	248487	74.11	3.20	16.77	72.10	75.30
	2012	13	ES	52766	54.81	3.31	6.25	51.20	54.90
	2012	18	BH	233088	38.21	3.00	16.05	37.10	39.40
	2012	18	BH	120619	46.91	3.75	7.90	44.90	47.40
	2012	18	ES	254573	34.91	3.29	6.04	32.70	37.60
**β ratio**	2011	18	BH	166745	23.61	8.10	16.06	23.20	24.40
	2011	18	BH	120619	47.41	4.20	7.91	44.90	47.60
	2011	18	BH	15694	53.51	3.03	5.29	53.20	55.80
	2012	18	BH	215808	24.31	3.10	5.69	23.90	27.10
	2012	18	BH	120619	47.41	4.22	8.34	45.10	47.60
	2012	18	BH	15694	53.51	3.23	6.09	53.20	55.40

Locations on linkage groups (LGs) of the BH and ES genetic maps, and contributions of the putative QTLs that control sugar- and acid-related traits, which were identified in at least two of three successive years (2011, 2012 and 2013). The locus is the marker showing the strongest association with the trait. The location of markers is given in cM, quoted from the top of each linkage group. *R* ^2^ represents the individual contribution of one QTL to the variation in a trait, and LOD is the logarithm of the odds ratio. SSC and β ratio represent soluble solids content and the tartaric acid-to-malic acid ratio, respectively. Traits in bold had QTLs detected in two years.

The number of QTLs identified for each trait varied between one and six, reflecting the quantitative nature of these traits, although no QTLs were detected for tartaric acid or titratable acidity. The QTLs that were identified were located within 13 of the 19 LGs. They each accounted for 5.28–17.31% of the total phenotypic variance in each trait.

Five QTLs for fructose were found in LG04, LG11, LG14, and LG17 of the ES map, each accounting for 5.58–9.71% of total variance. Three QTLs controlling glucose were found in LG14 of the ES map, contributing 6.04–8.16% of the variance. The QTLs for total sugar overlapped with those for fructose and/or glucose in LG14 of the ES map, individually contributing 5.85–8.37% of the variance. The QTL for SSC in LG14 of the ES map was the same as that for fructose, glucose and total sugar. A QTL for SSC was also identified in LG18 of the BH map, which explained 6.03% of the variance. A QTL for the glucose-to-fructose ratio and the α ratio, which was identified in LG03 and LG09 of the BH map, did not overlap with any of those for the individual sugars. Another QTL for the glucose-to-fructose ratio was found in LG02. QTLs for glucose-to-fructose ratio were also found in LG07 and LG17, on the ES map.

Malic acid, total acid and β ratio each had two to six QTLs in LG18 of the BH map, and there was another QTL for the β ratio in LG13 and LG18 of the ES map. There was also one QTL for both malic acid and total acid in LG06 of the BH map, which contributed 16.77–17.31% of the variance. However, no QTL could be identified for tartaric acid or titratable acidity.

### Candidate gene identification

In total, we identified 499 genes underlying the 19 QTLs of the BH map, and 724 genes underlying the 19 QTLs of the ES map. Of these, 835 (68.3%) were annotated and classified. However, only two QTLs (for malic acid, total acid and β ratio on LG18 of BH map) were stable across years (having been observed in two years). We therefore henceforward focused only on the candidate genes located within the confidence intervals of these two QTLs. For these two QTLs, 134 candidate genes were found. They were unevenly distributed, with 106 (22.8–26.8 cM) for one and 28 (45.5–47.6 cM) for the other QTL. Fifty of these genes were catalogued as having an “unknown protein function”, and the others were classified into six major groups, namely cell, glycolysis, protein, RNA, TCA/org transformation, and transport. Of the 134 candidate genes, 10 that were probably related to TCA, acid metabolism or transport were listed, mainly (but not exclusively) based on their biological function as described in model plant species such as *Arabidopsis*, rice and poplar (Table 4).

**Table 4:** **Genes in LG18 that may participate in acid regulation**

**Groups**	**Position**	**Gene ID**	**Gene symbol**	**Description**	**References**
Cell	5462823-5465587	GSVIVT01009139001	CYCD4;1	CYCLIN D4;1	[87]
Glycolysis	5505643-5516683	GSVIVT01009147001	PGI,PGI1	phosphoglucose isomerase 1	[88]
protein	6442873-6452788	GSVIVT01009228001	ATCIPK8,CIPK8,PKS11,SnRK3.13	CBL-interacting protein kinase 8	[89]
protein	6652256-6660443	GSVIVT01009251001	ATDBR1,DBR1	debranching enzyme 1	[90]
RNA	6526891-6532321	GSVIVT01009238001	IAA9	indole-3-acetic acid inducible 9	[91,92]
TCA/org transformation	5663288-5667315	GSVIVT01009165001	ATBCA5,BCA5	beta carbonic anhydrase 5	[93]
transport	6771378-6774993	GSVIVT01009260001	AAP6	amino acid permease 6	[94]
transport	5605382-5606712	GSVIVT01009152001	ATPUMP5,DIC1,UCP5	uncoupling protein 5	[86]
transport	6688299-6690573	GSVIVT01009253001	ZF14	MATE efflux family protein	[80-85]
transport	10039446-10043834	GSVIVT01009629001		MATE efflux family protein	[80-85]

## Discussion

### Phenotypic evaluation

The grape berries we analyzed displayed similar substantial variation in sugar and acid concentration across three successive years, which supports previous results showing that sugar and acid concentrations of grapes vary significantly by year [1]. For the study period, fructose, glucose, total sugar, tartaric acid, malic acid, and total acid concentration ranges were 7.5–136.7, 7.8–154.4, 15.3–291.9, 1.5–17.2, 0.8–21.3, and 4.9–34.7 mg·mL^−1^, respectively. These ranges were greater than those found in other *Vitis* populations [49,69]. The reported ranges for fructose, glucose, and total sugar concentrations for these populations are 36.2–111.9, 38.5–104.4, and 78.9–216.3 mg·mL^−1^, respectively, and those for tartaric acid, malic acid, and total acid concentrations are 1.1–6.0, 0.6–8.3 and 2.1–11.8 mg·mL^−1^, respectively. Phenotypic correlations between sugar and acid concentrations were also relatively stable across the three years, although these may be affected by environmental factors.

### Genetic map

Although genetic maps for grape cultivars have developed greatly in recent years, the number of markers in the LGs in existing maps is still generally less than 1 000, and some of the mapped markers have no sequence information. We recently identified 1 814 high-quality SNP markers for a population of ‘Z180’ (1 212 markers) × ‘Beihong’ (759 markers) [33]. In this study we used the same procedure to construct the genetic map, and the density of the resultant linkage map was similarly high. In total we identified 1 826 SNP markers, 621 of which were mapped on the female BH genetic map, and 696 on the male ES map. The difference between the number of markers we identified in this study and in the earlier one may be related to the different F1 population. On the BH map, the average size of LGs was 81.76 cM, ranging from 25.04 cM (LG07) to 112.14 cM (LG19). On the ES map, the average size was 72.69 cM, ranging from 22.83 cM (LG05) to 104.33 cM (LG04). There were 17 and 12 marker-free regions longer than 10 cM on the BH map (LG02, 03, 04, 05, 09, 11, 14, 16, 19) and the ES map (LG02, 04, 06, 09, 10, 11, 14, 15, 18), respectively.

The total physical size of the grape genome is ~470 Mb [66,70]. In most regions of the parental genetic and physical maps (for *V. vinifera*), the markers occurred in the same order, but not in all the chromosome regions. This indicates that on one hand the genome is well conserved among grape species, but that some changes in marker order have occurred during speciation. In this study, the maternal parent, BH, is bred from *V. vinifera* and *V. amurensis*, and the paternal parent, ES, is bred from *V. labrusca* × *V. riparia* and *V. vinifera*. Differences in the order of markers on some chromosomes have presumably resulted from different micro-structures on chromosomes in the various species. Alternatively, these differences might have arisen because of possible errors in mapping causing small inversions in marker order.

### QTL detection

QTLs were analyzed separately for each of the traits on the parental maps for each of the three years, but were inconsistently detected. Some minor QTLs were detected only in a single year, such as a glucose QTL in LG14, for which *R*^*2*^ = 6.04–6.18%, and a total sugar QTL in LG14, with *R*^*2*^ = 5.85–6.72%. Other QTLs that contributed strongly to total variance were also detected in only one year, e.g. an SSC QTL in LG14 in 2011, for which *R*^*2*^ = 11.42%, and a malic acid QTL in LG06 in 2011, with *R*^*2*^ = 17.31%. In some cases, no QTLs were detected for a trait in a specific year, e.g. malic acid and total acid in 2013. Similar instability of QTLs across years has been widely reported for grapes [14,42,71], and also for other fruit tree species [52,57,72-74]. In contrast to crops such as maize, soya and rice, in which there may be many plants and biological replications of each genotype in one growth environment, there was only one vine per genotype in our trial. Phenotypic value assessment is potentially subject to bias which would increase the likelihood of error and affect the QTL analysis, with possible results including underestimated LOD values and overlap between QTLs across years. Furthermore, variation in climatic factors (such as rainfall and temperature) between years could bias assessment of fruit maturity, which would affect phenotypic evaluations. The observed low repeatability of QTL detection may thus have been exacerbated by the lack of replicate vines and the potential inconsistency in assessment of maturity. Addressing these problems in QTL studies on fruit species is difficult, however.

The percentage of variation explained by each QTL was small, and varied between 5.29% and 17.31%. This is consistent with the low *R*^*2*^ values previously reported for some grape agronomic traits. Fanizza *et al.* [42] found that a QTL controlling berry weight had *R*^*2*^ = 19%, but QTLs controlling the number of clusters per vine, cluster weight, number of berries per cluster, and berry weight had substantially lower *R*^*2*^ values (1.2–10%). Similarly, Viana *et al.* [50] found that most QTLs accounted for less than 5.5% of the variance. QTLs with high *R*^*2*^ values have generally been found to be related to properties such as veraison time/period, anthocyanin content (up to 48–62%) [47], and seed dry/fresh weight (up to 91.4%) [14,71]. It seems that agronomic traits, including sugar and acid concentration, are generally controlled by numerous QTLs, each with small effects. This might be due to the quantitative nature of these traits, as well as complicated metabolic pathways and regulatory networks.

Heritability for most traits is generally less than 50%, so the heritability associated with each QTL is a small fraction of this [75]. The more QTLs there are in the population, the smaller their individual contribution and the more difficult they are to detect [75]. As a result, precise map construction may be challenging, and maps may include some QTLs with very small *R*^*2*^ values. Furthermore, the number of QTLs detected and the phenotypic variance they explain might be biased because of the limitations of the experiment itself, such as small sample size (as the effectiveness of marker loci increases with the number of individuals in a population) [76].

To our knowledge, no QTLs controlling the production of individual sugars and acids in grape berries have previously been identified. Viana *et al.* [50] reported one QTL in LG03 for SSC, one QTL in each of LG06, 13 and 19 for titratable acidity (% tartaric acid), and one QTL in each of LG01, 06, 11, 13 and 16 for pH, based on results for one year. We did not detect a QTL in LG03 for SSC in any of the three years of our study, or any QTLs for titratable acidity. This discrepancy between results may result from different genetic determinants of trait variation in the populations studied. Another cause might be differences in sampling strategies and the methods used for measuring traits. In this study, a QTL for malic acid in LG06 positioned at 74.11 cM explained a relatively large amount of variance (17.31%). Viana *et al.* [50] reported a QTL in LG06 positioned at 0.00 cM for pH, which explained 10.34% of variance. Although they were not the same QTL, these two regions might be worth exploring for genes controlling the quality of fruit acidity.

### QTLs co-location

For breeding purposes, it is worth examining QTLs that are co-located. With respect to individual sugars, a QTL in LG14 affected both fructose and glucose, which explained the high correlation between them (*r* = 0.93). However, this QTL was detected only in 2011. In peaches, three QTLs, located in three different LGs are related to both glucose and fructose concentration [52]. The co-location of QTLs controlling fructose and glucose probably indicates a unique gene with a pleiotropic effect, or genes with close linkage, because glucose and fructose are absent from phloem sap and in grape berries are synthesized concurrently by sucrose hydrolysis [77]. The QTL in LG14 is potentially promising to work with to increase sugar concentrations, which may be beneficial for wine-making. Further study on candidate functional genes within the confidence intervals of this QTL may help to assess the mechanism for controlling hexose metabolism. The QTL for total sugar in LG14 overlapped with the QTL for fructose and glucose, which is probably related to the fact that these individual sugars contribute to total sugar. Similarly, a QTL for SSC in LG14 overlapped with one for fructose and glucose. However, the fact that the QTLs for the glucose-to-fructose ratio were not co-located with QTLs for either glucose or fructose is difficult to explain. With respect to the acid-related traits, QTLs for total acid and β ratio were co-located with those for malic acid, suggesting that malic acid contributes greatly to total acid and β ratio.

An important problem encountered when breeding for improved traits is negative correlations between favorable traits. For instance, as our results confirmed, malic acid and fructose are negatively correlated in 98 grape cultivars [1]. In some cases, this was caused by co-located QTLs with opposite horticultural effects. In tomatoes, fruit size and soluble sugar concentration are often negatively correlated, and the QTLs controlling them are located in the same LG [78,79]. In this study, although fructose, glucose, total sugar and SSC were negatively correlated with tartaric acid, malic acid, total acid and titratable acidity, in six LGs (01, 04, 11, 14, 17, 18) there were 15 QTLs related to sugars, and in three LGs (06, 13, 18) there were 11 QTLs related to acids. Viana *et al.* [50] also reported different LGs for sugar- and acid-related traits: one QTL in LG3 for SSC, and one QTL for pH and titratable acidity in each of LG01, 06, 11, 13, 16 and 19. The non-co-location of QTLs controlling sugar and acid concentration is favorable from the perspective of breeding. However, it is likely that some QTLs that have not yet been detected contribute to the remaining unexplained variance, and these should not be ignored.

### Candidate genes for acid regulation

Of 134 genes located within the confidence intervals of the two QTLs controlling malic acid, total acid, and β ratio in LG18, 11 were probably involved in acid metabolism [80-94]. For example, beta carbonic anhydrase 5 is related to TCA/org transformation, and malic acid is involved in this process. Numerous studies reveal that the MATE family plays a key role in malate efflux from root apices in many plant species, including *Arabidopsis*, maize (*Zea mays*), wheat, rice (*Oryza sativa*), and rice bean (*Vignaumbellata*) [80-85]. Uncoupling protein 5 (DIC1) belongs to the mitochondrial carrier protein family. The *Arabidopsis* DIC proteins transport a wide range of dicarboxylic acids including malate, oxaloacetate and succinate [86], and these proteins might function as malate/oxaloacetate shuttles, which provide other cell components with reducing equivalents [86]. Expression levels of or changes in these candidate genes could be evaluated in further transcriptomic and gene-directed studies. The identification of the most relevant genes would help to reveal the molecular mechanisms operating in grape cultivars and could have a large impact on future breeding efforts.

## Conclusions

The genetic map we have constructed for a winemaking grape cross is potentially a high-density, high-quality map, which could be used for QTL detection, genome comparison, and sequence assembly. In total, we mapped 1 254 markers with 60 bp sequences on the integrated map. These markers can be used as anchors to compare genetic and physical maps. This may facilitate the improved use of grape genomic resources. Moreover, this genetic map of a cross with a complex parentage, (*V. vinifera* × *V. amurensis*) × ((*V. labrusca* × *V. riparia*) × *V. vinifera*), will help to broaden our understanding of the grape genome. However, the stability and accuracy of QTLs are also affected by environmental factors. In our study, year and climatic conditions were the most important source of variability. Thus, controlling environmental factors can increase the likelihood that an observed phenotypic value is both accurate and repeatable.

Several QTLs controlling berry sugar and acid traits were detected in different LGs, suggesting that these traits are influenced by several genes that control different aspects of complex metabolic pathways. For example, we have identified a set of 10 candidate genes underlying the QTLs that are potentially related to malic acid. We anticipate that we will soon be able to narrow down these regions to the point where effects can be ascribed to specific genes. In addition, studies based on transcriptomics, proteomics, and metabolomics would help us to achieve a more accurate understanding of the molecular parameters involved in berry sugar and acid regulation. The long-term objective of this research is to provide information on the genetic basis of these traits, and to facilitate the selection of varieties to improve sugar and acid quality.

## Additional files

Additional file 1: Figure S1. Distribution of traits of F1 population derived from the cross ‘Beihong’ (BH) × ‘E.S.7-11-49’ (ES) in 2011 and 2012. Additional file 2: Figure S2. Genetic maps and QTL locations for ‘Beihong’ (BH, maternal parent) and ‘E.S.7-11-49’ (ES, paternal parent). Additional file 3: Table S1. SNP-based markers and genetic positions on the 19 linkage groups (LGs). Additional file 4: Figure S3. Positions of SNP-based markers on genetic maps, and their physical positions on the reference genome.
