# Supplementary material for: Construction of a high-density genetic map and QTLs mapping for sugars and acids in grape berries
Source: BMC Plant Biol. 2015 Feb 3;15:28. doi: 10.1186/s12870-015-0428-2 (PMC4329212; doi:10.1186/s12870-015-0428-2)

## Slide 1
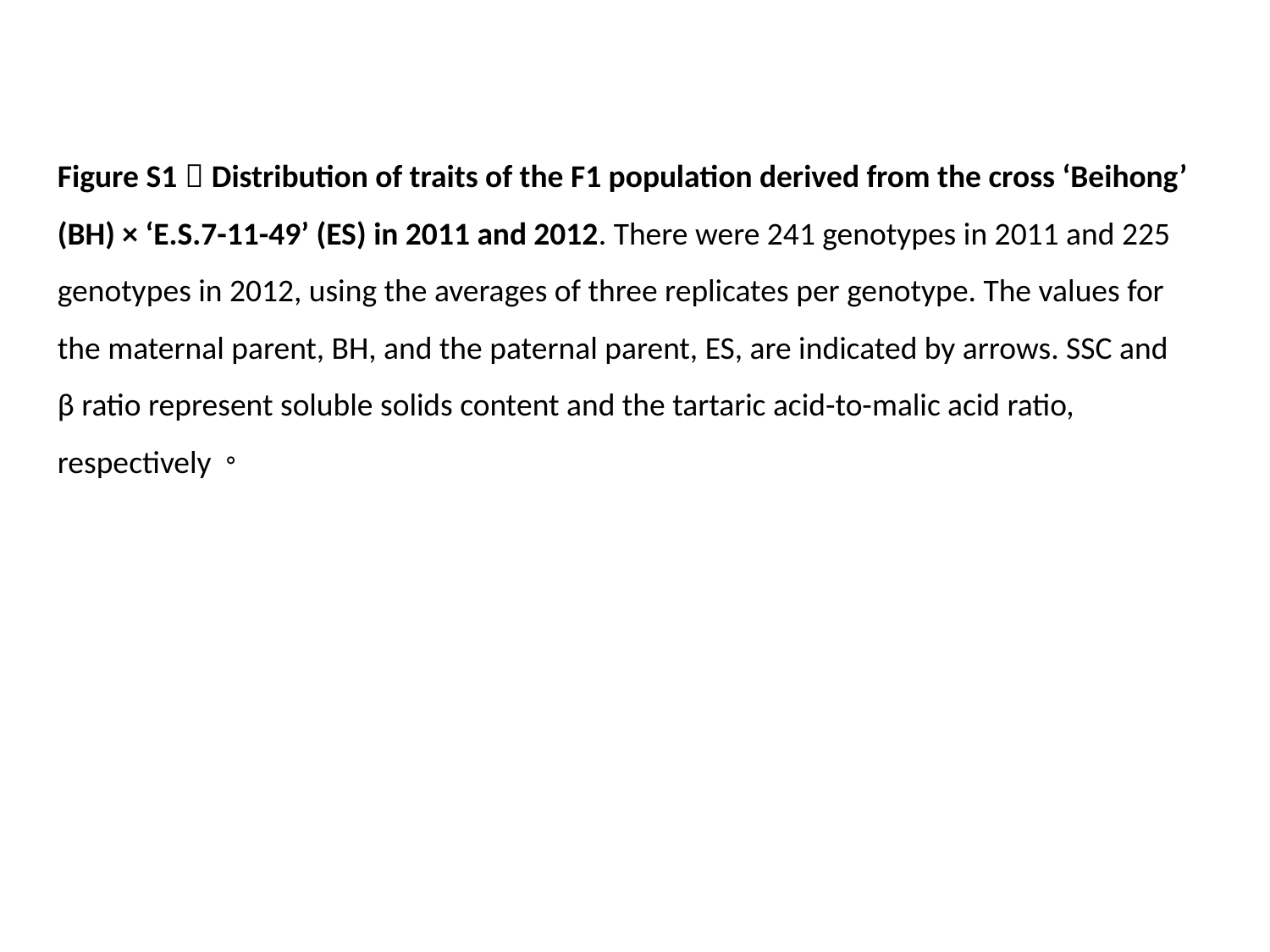

Figure S1．Distribution of traits of the F1 population derived from the cross ‘Beihong’ (BH) × ‘E.S.7-11-49’ (ES) in 2011 and 2012. There were 241 genotypes in 2011 and 225 genotypes in 2012, using the averages of three replicates per genotype. The values for the maternal parent, BH, and the paternal parent, ES, are indicated by arrows. SSC and β ratio represent soluble solids content and the tartaric acid-to-malic acid ratio, respectively。

## Slide 2
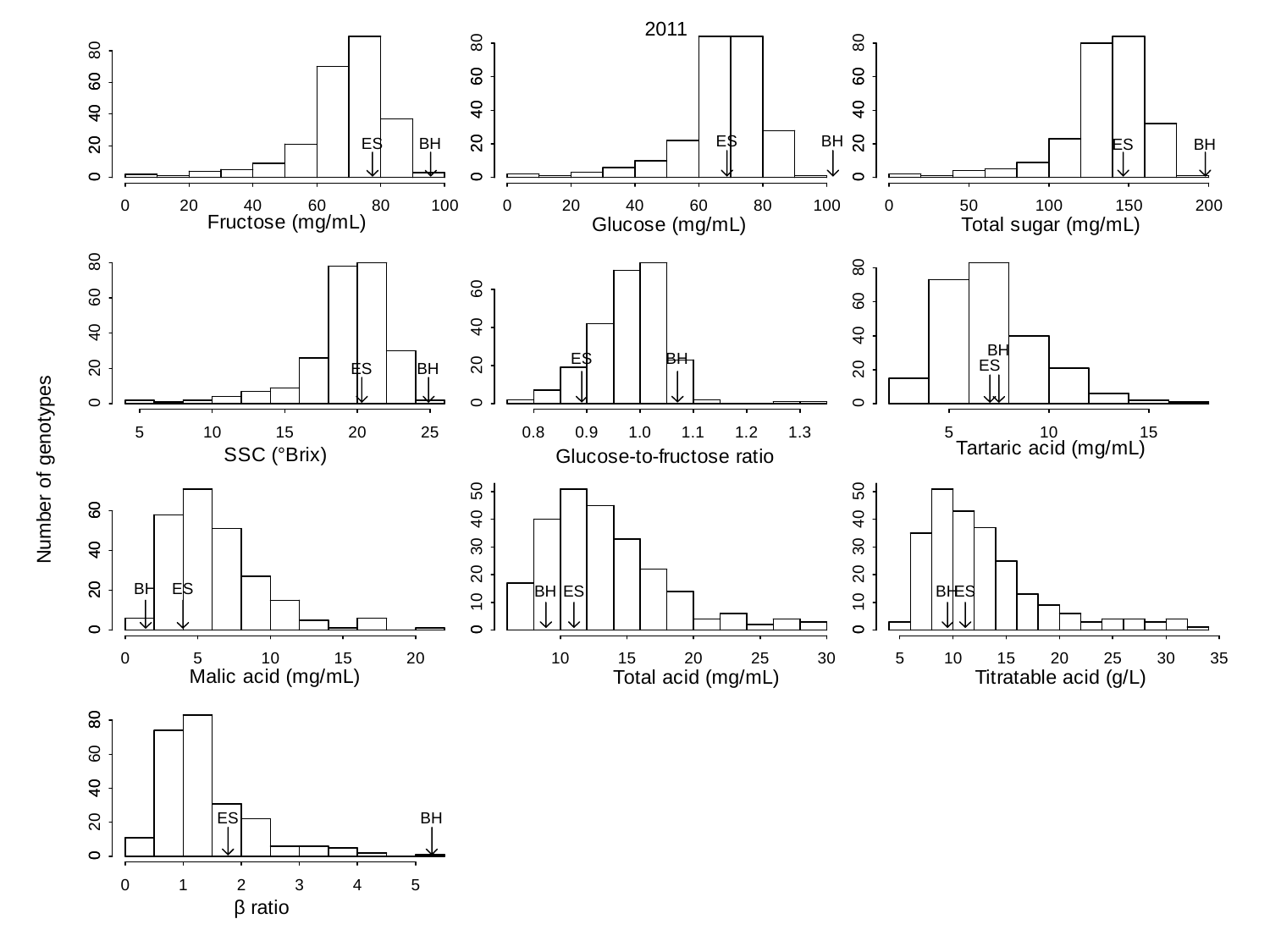

## Slide 3
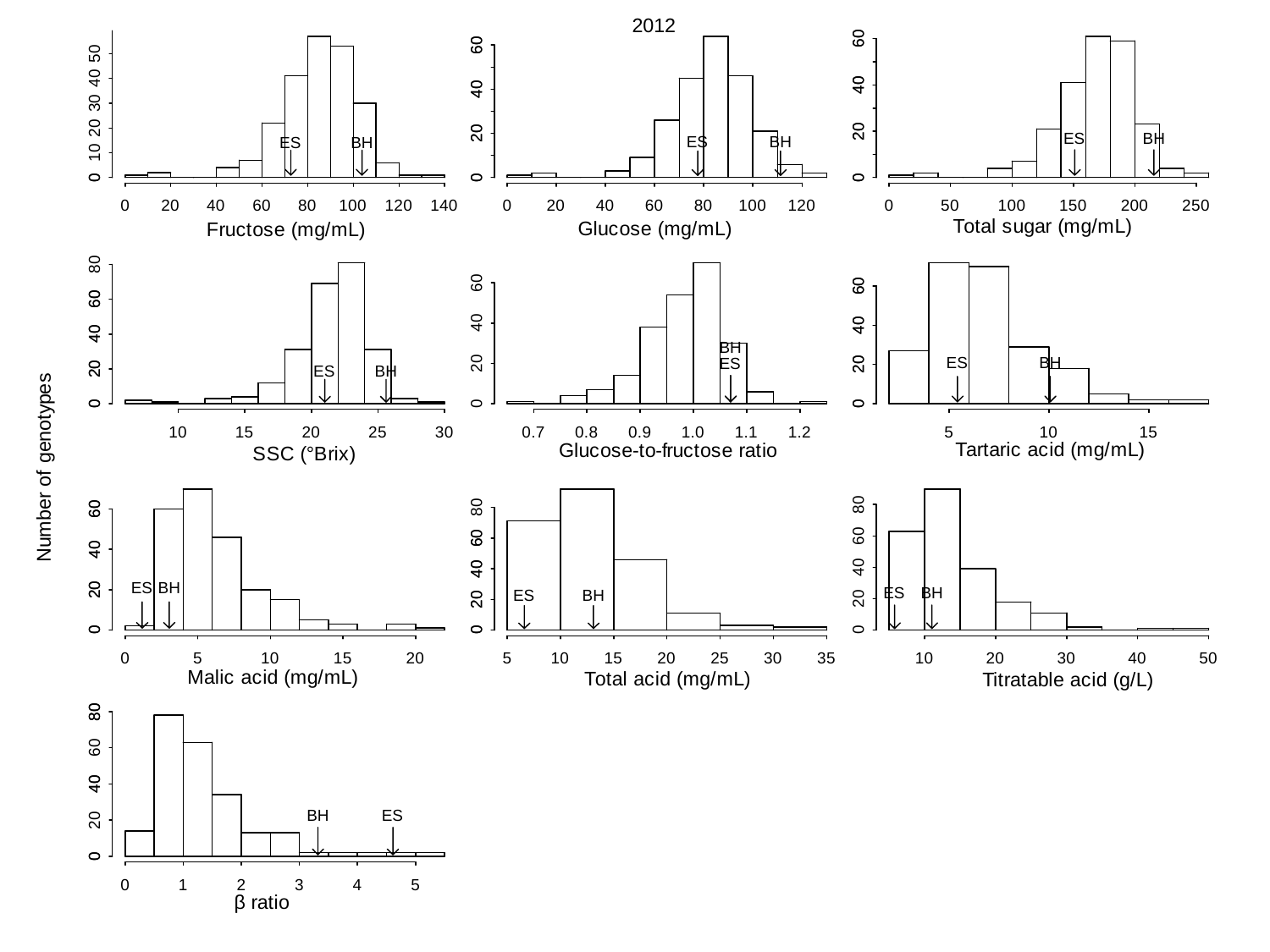

Supplement: Additional file 1: Figure S1. — Distribution of traits of F1 population derived from the cross ‘Beihong’ (BH) × ‘E.S.7-11-49’ (ES) in 2011 and 2012. [file 12870_2015_428_MOESM1_ESM.pptx]
