# Supplementary material for: Construction of a high-density genetic map and QTLs mapping for sugars and acids in grape berries
Source: BMC Plant Biol. 2015 Feb 3;15:28. doi: 10.1186/s12870-015-0428-2 (PMC4329212; doi:10.1186/s12870-015-0428-2)
Supplement: Additional file 2: Figure S2. — Genetic maps and QTL locations for ‘Beihong’ (BH, maternal parent) and ‘E.S.7-11-49’ (ES, paternal parent). [file 12870_2015_428_MOESM2_ESM.pptx]

## Slide 1
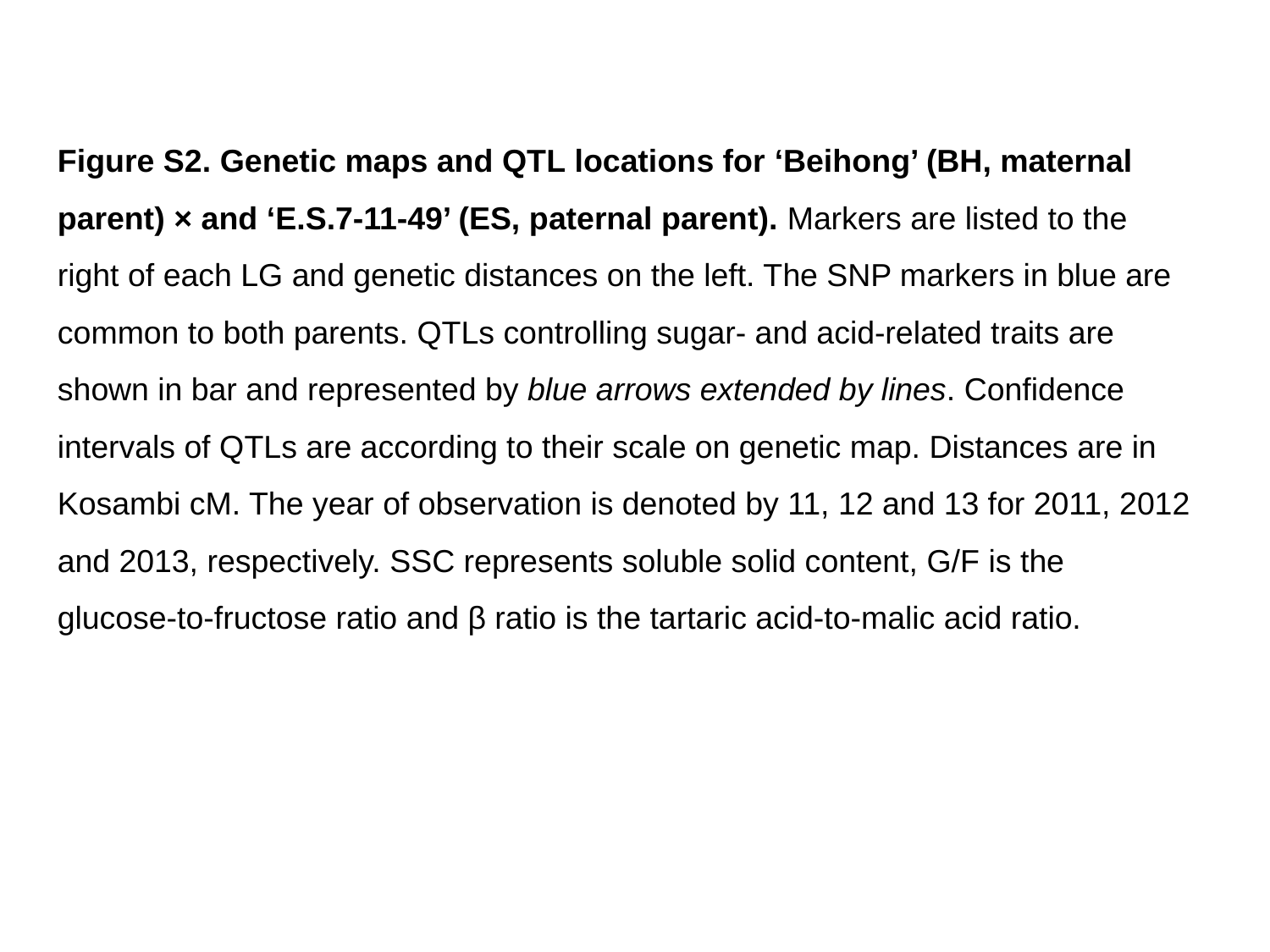

Figure S2. Genetic maps and QTL locations for ‘Beihong’ (BH, maternal parent) × and ‘E.S.7-11-49’ (ES, paternal parent). Markers are listed to the right of each LG and genetic distances on the left. The SNP markers in blue are common to both parents. QTLs controlling sugar- and acid-related traits are shown in bar and represented by blue arrows extended by lines. Confidence intervals of QTLs are according to their scale on genetic map. Distances are in Kosambi cM. The year of observation is denoted by 11, 12 and 13 for 2011, 2012 and 2013, respectively. SSC represents soluble solid content, G/F is the glucose-to-fructose ratio and β ratio is the tartaric acid-to-malic acid ratio.

## Slide 2
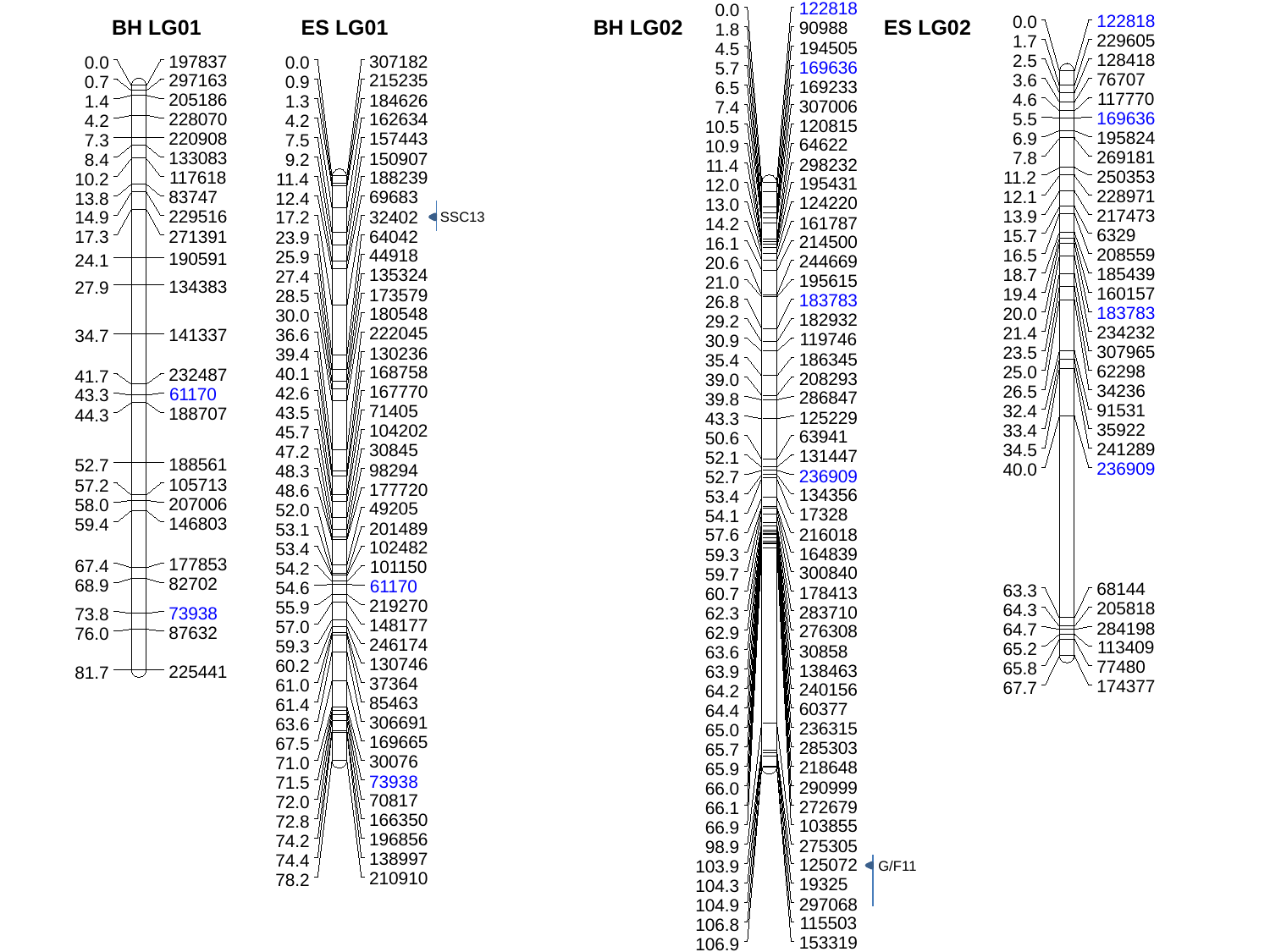

122818
0.0
90988
1.8
194505
4.5
169636
5.7
169233
6.5
307006
7.4
120815
10.5
64622
10.9
298232
11.4
195431
12.0
124220
13.0
161787
14.2
214500
16.1
244669
20.6
195615
21.0
183783
26.8
182932
29.2
119746
30.9
186345
35.4
208293
39.0
286847
39.8
125229
43.3
63941
50.6
131447
52.1
236909
52.7
134356
53.4
17328
54.1
216018
57.6
164839
59.3
300840
59.7
178413
60.7
283710
62.3
276308
62.9
30858
63.6
138463
63.9
240156
64.2
60377
64.4
236315
65.0
285303
65.7
218648
65.9
290999
66.0
272679
66.1
103855
66.9
275305
98.9
125072
103.9
19325
104.3
297068
104.9
115503
106.8
153319
106.9
G/F11
122818
0.0
229605
1.7
128418
2.5
76707
3.6
117770
4.6
169636
5.5
195824
6.9
269181
7.8
250353
11.2
228971
12.1
217473
13.9
6329
15.7
208559
16.5
185439
18.7
160157
19.4
183783
20.0
234232
21.4
307965
23.5
62298
25.0
34236
26.5
91531
32.4
35922
33.4
241289
34.5
236909
40.0
68144
63.3
205818
64.3
284198
64.7
113409
65.2
77480
65.8
174377
67.7
BH LG01
ES LG01
BH LG02
ES LG02
197837
0.0
297163
0.7
205186
1.4
228070
4.2
220908
7.3
133083
8.4
117618
10.2
83747
13.8
229516
14.9
271391
17.3
190591
24.1
134383
27.9
141337
34.7
232487
41.7
61170
43.3
188707
44.3
188561
52.7
105713
57.2
207006
58.0
146803
59.4
177853
67.4
82702
68.9
73938
73.8
87632
76.0
225441
81.7
307182
0.0
215235
0.9
184626
1.3
162634
4.2
157443
7.5
150907
9.2
188239
11.4
69683
12.4
32402
17.2
64042
23.9
44918
25.9
135324
27.4
173579
28.5
180548
30.0
222045
36.6
130236
39.4
168758
40.1
167770
42.6
71405
43.5
104202
45.7
30845
47.2
98294
48.3
177720
48.6
49205
52.0
201489
53.1
102482
53.4
101150
54.2
61170
54.6
219270
55.9
148177
57.0
246174
59.3
130746
60.2
37364
61.0
85463
61.4
306691
63.6
169665
67.5
30076
71.0
73938
71.5
70817
72.0
166350
72.8
196856
74.2
138997
74.4
210910
78.2
SSC13

## Slide 3
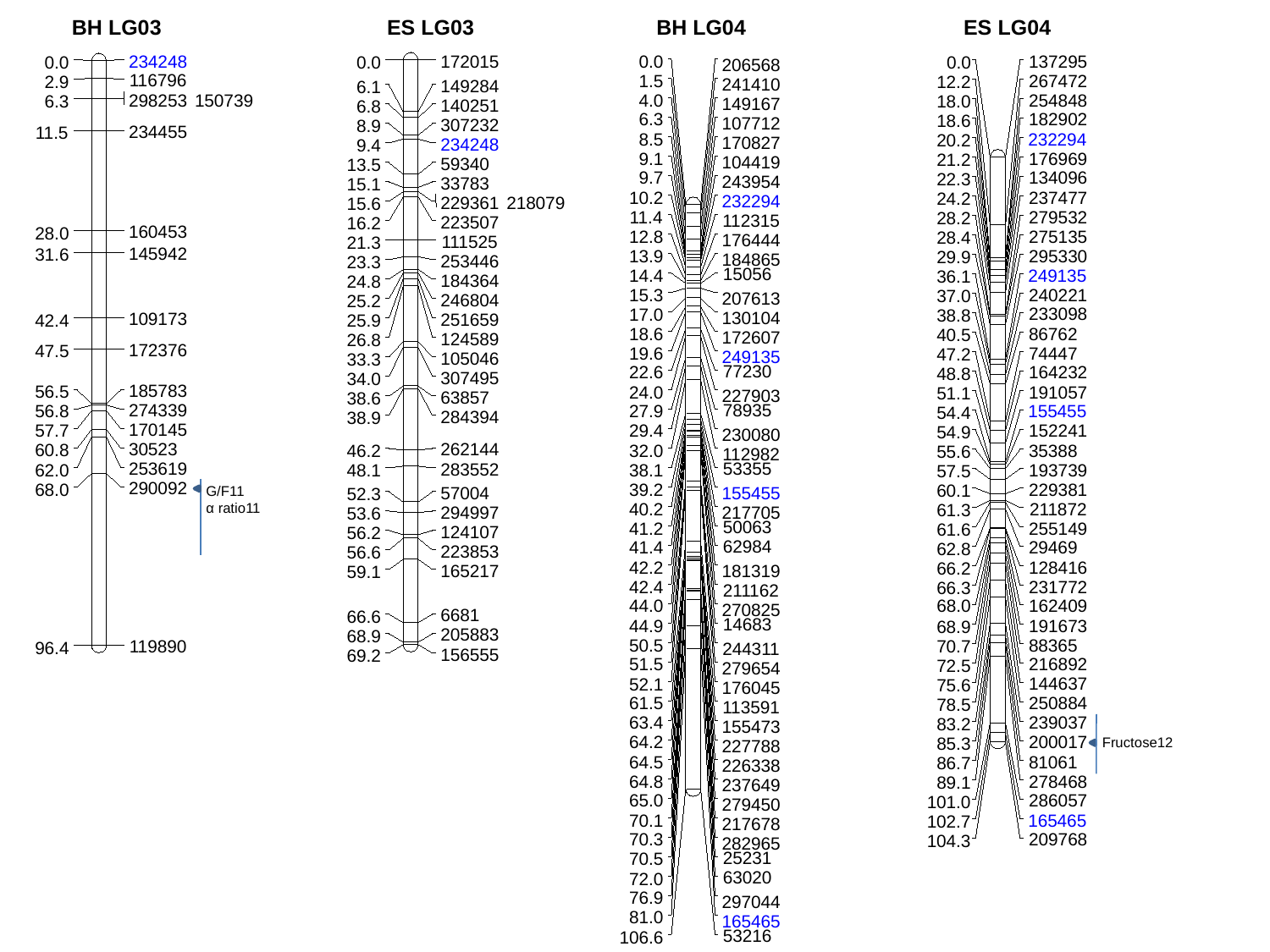

BH LG03
ES LG03
BH LG04
ES LG04
234248
0.0
116796
2.9
298253
150739
6.3
234455
11.5
160453
28.0
145942
31.6
109173
42.4
172376
47.5
185783
56.5
274339
56.8
170145
57.7
30523
60.8
253619
62.0
290092
68.0
119890
96.4
172015
0.0
149284
6.1
140251
6.8
307232
8.9
234248
9.4
59340
13.5
33783
15.1
229361
218079
15.6
223507
16.2
111525
21.3
253446
23.3
184364
24.8
246804
25.2
251659
25.9
124589
26.8
105046
33.3
307495
34.0
63857
38.6
284394
38.9
262144
46.2
283552
48.1
57004
52.3
294997
53.6
124107
56.2
223853
56.6
165217
59.1
6681
66.6
205883
68.9
156555
69.2
0.0
206568
1.5
241410
4.0
149167
6.3
107712
8.5
170827
9.1
104419
9.7
243954
10.2
232294
11.4
112315
12.8
176444
13.9
184865
15056
14.4
15.3
207613
17.0
130104
18.6
172607
19.6
249135
77230
22.6
24.0
227903
78935
27.9
29.4
230080
32.0
112982
53355
38.1
39.2
155455
40.2
217705
50063
41.2
62984
41.4
42.2
181319
42.4
211162
44.0
270825
14683
44.9
50.5
244311
51.5
279654
52.1
176045
61.5
113591
63.4
155473
64.2
227788
64.5
226338
64.8
237649
65.0
279450
70.1
217678
70.3
282965
25231
70.5
63020
72.0
76.9
297044
81.0
165465
53216
106.6
137295
0.0
267472
12.2
254848
18.0
182902
18.6
232294
20.2
176969
21.2
134096
22.3
237477
24.2
279532
28.2
275135
28.4
295330
29.9
249135
36.1
240221
37.0
233098
38.8
86762
40.5
74447
47.2
164232
48.8
191057
51.1
155455
54.4
152241
54.9
35388
55.6
193739
57.5
229381
60.1
211872
61.3
255149
61.6
29469
62.8
128416
66.2
231772
66.3
162409
68.0
191673
68.9
88365
70.7
216892
72.5
144637
75.6
250884
78.5
239037
83.2
200017
85.3
81061
86.7
278468
89.1
286057
101.0
165465
102.7
209768
104.3
Fructose12
G/F11
α ratio11

## Slide 4
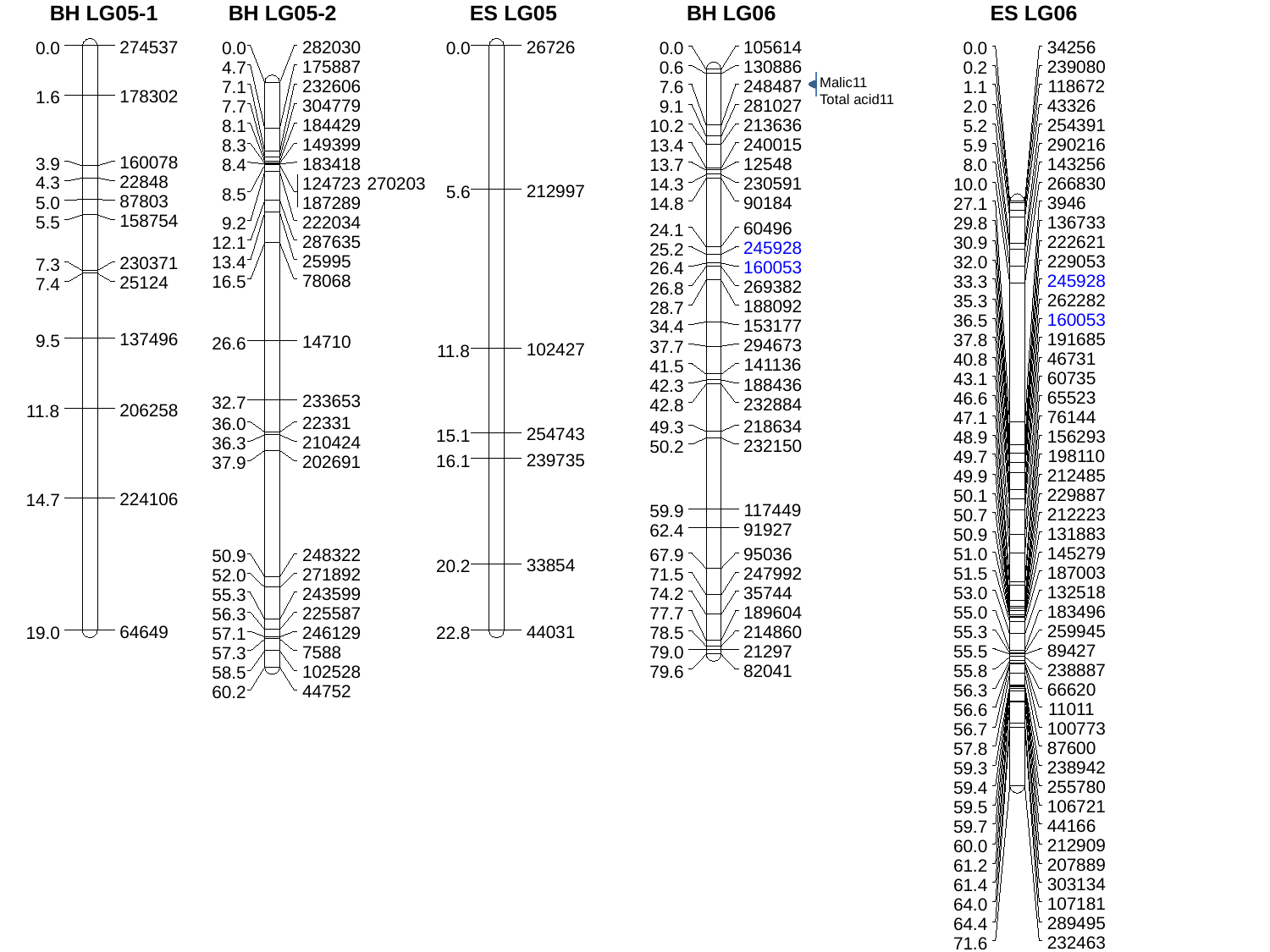

BH LG05-1
BH LG05-2
ES LG05
BH LG06
ES LG06
274537
0.0
178302
1.6
160078
3.9
22848
4.3
87803
5.0
158754
5.5
230371
7.3
25124
7.4
137496
9.5
206258
11.8
224106
14.7
64649
19.0
282030
0.0
175887
4.7
232606
7.1
304779
7.7
184429
8.1
149399
8.3
183418
8.4
124723
270203
8.5
187289
222034
9.2
287635
12.1
25995
13.4
78068
16.5
14710
26.6
233653
32.7
22331
36.0
210424
36.3
202691
37.9
248322
50.9
271892
52.0
243599
55.3
225587
56.3
246129
57.1
7588
57.3
102528
58.5
44752
60.2
26726
0.0
212997
5.6
102427
11.8
254743
15.1
239735
16.1
33854
20.2
44031
22.8
105614
0.0
130886
0.6
248487
7.6
281027
9.1
213636
10.2
240015
13.4
12548
13.7
230591
14.3
90184
14.8
60496
24.1
245928
25.2
160053
26.4
269382
26.8
188092
28.7
153177
34.4
294673
37.7
141136
41.5
188436
42.3
232884
42.8
218634
49.3
232150
50.2
117449
59.9
91927
62.4
95036
67.9
247992
71.5
35744
74.2
189604
77.7
214860
78.5
21297
79.0
82041
79.6
Malic11
Total acid11
34256
0.0
239080
0.2
118672
1.1
43326
2.0
254391
5.2
290216
5.9
143256
8.0
266830
10.0
3946
27.1
136733
29.8
222621
30.9
229053
32.0
245928
33.3
262282
35.3
160053
36.5
191685
37.8
46731
40.8
60735
43.1
65523
46.6
76144
47.1
156293
48.9
198110
49.7
212485
49.9
229887
50.1
212223
50.7
131883
50.9
145279
51.0
187003
51.5
132518
53.0
183496
55.0
259945
55.3
89427
55.5
238887
55.8
66620
56.3
11011
56.6
100773
56.7
87600
57.8
238942
59.3
255780
59.4
106721
59.5
44166
59.7
212909
60.0
207889
61.2
303134
61.4
107181
64.0
289495
64.4
232463
71.6

## Slide 5
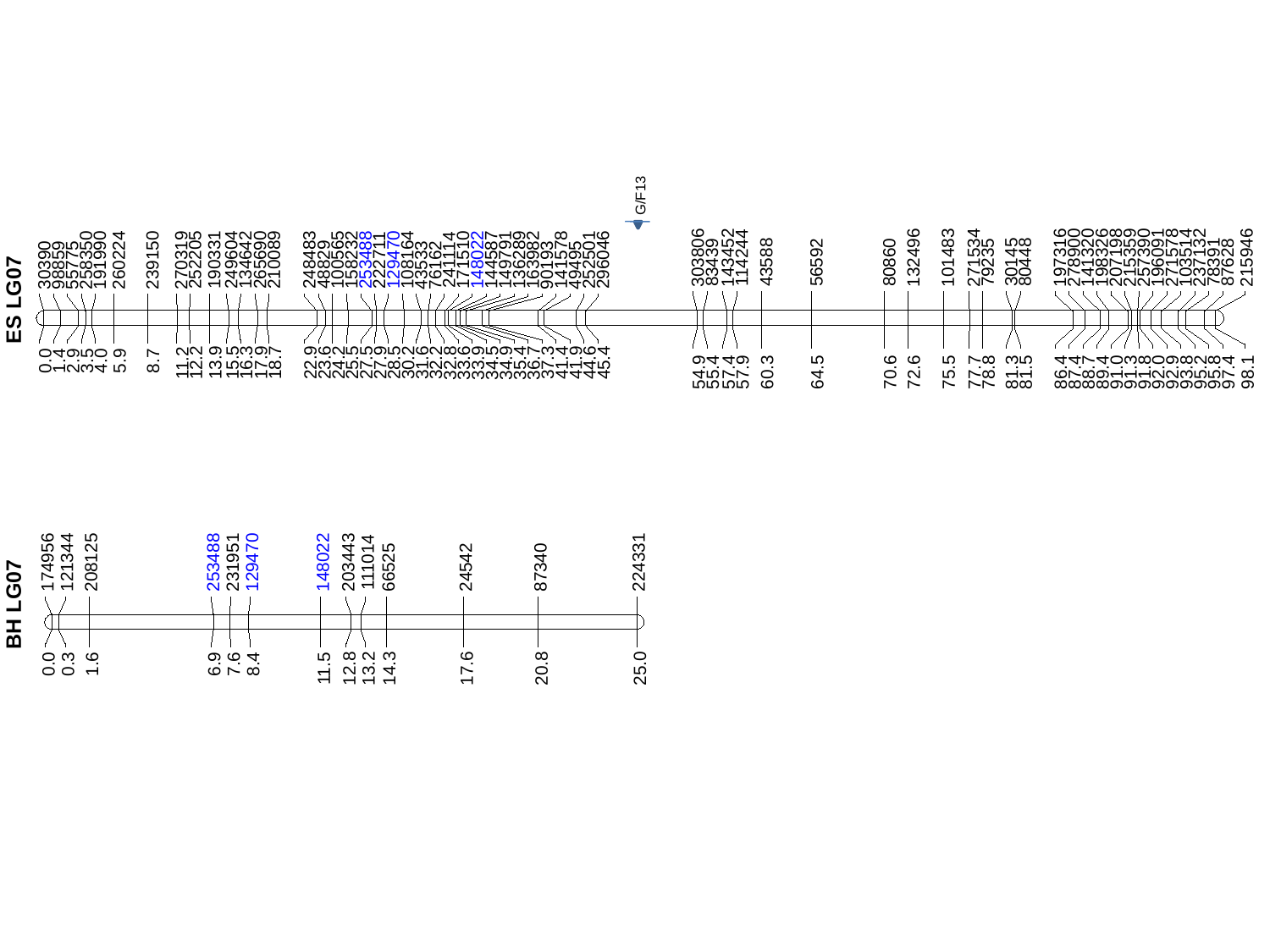

30390
0.0
98859
1.4
55775
2.9
258350
3.5
191990
4.0
260224
5.9
239150
8.7
270319
11.2
252205
12.2
190331
13.9
249604
15.5
134642
16.3
265690
17.9
210089
18.7
248483
22.9
48829
23.6
100565
24.2
158232
25.5
253488
27.5
222711
27.9
129470
28.5
108164
30.2
43533
31.6
76162
32.2
241114
32.8
171510
33.6
148022
33.9
144587
34.5
149791
34.9
136289
35.4
163982
36.7
90193
37.3
141578
41.4
49495
41.9
252501
44.6
296046
45.4
303806
54.9
83439
55.4
143452
57.4
114244
57.9
43588
60.3
56592
64.5
80860
70.6
132496
72.6
101483
75.5
271534
77.7
79235
78.8
30145
81.3
80448
81.5
197316
86.4
278900
87.4
141320
88.7
198326
89.4
207198
91.0
215359
91.3
257390
91.8
196091
92.0
271578
92.9
103514
93.8
237132
95.2
78391
95.8
87628
97.4
215946
98.1
G/F13
ES LG07
174956
0.0
121344
0.3
208125
1.6
253488
6.9
231951
7.6
129470
8.4
148022
11.5
203443
12.8
111014
13.2
66525
14.3
24542
17.6
87340
20.8
224331
25.0
BH LG07

## Slide 6
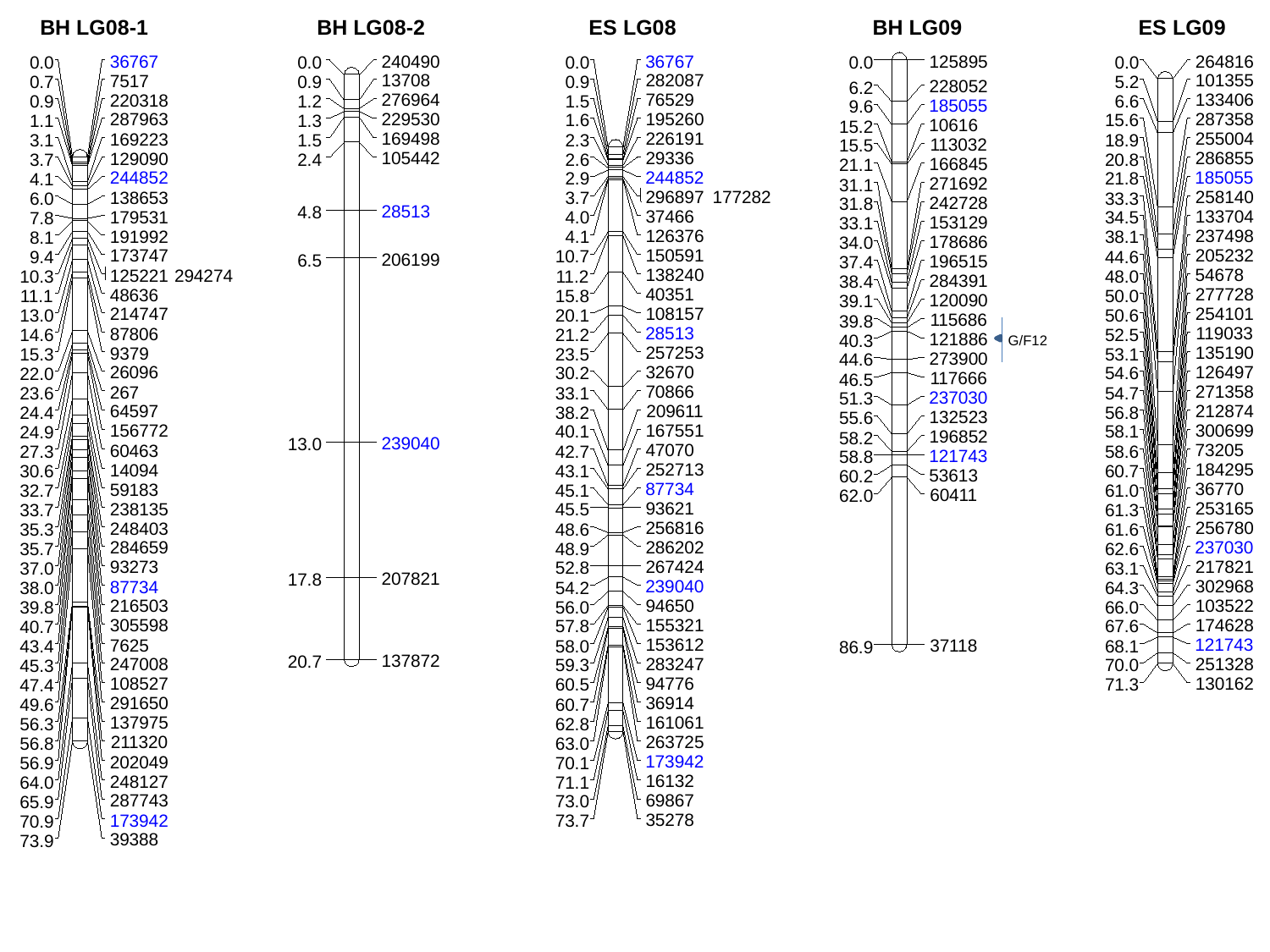

BH LG08-1
BH LG08-2
ES LG08
BH LG09
ES LG09
36767
0.0
7517
0.7
220318
0.9
287963
1.1
169223
3.1
129090
3.7
244852
4.1
138653
6.0
179531
7.8
191992
8.1
173747
9.4
125221
294274
10.3
48636
11.1
214747
13.0
87806
14.6
9379
15.3
26096
22.0
267
23.6
64597
24.4
156772
24.9
60463
27.3
14094
30.6
59183
32.7
238135
33.7
248403
35.3
284659
35.7
93273
37.0
87734
38.0
216503
39.8
305598
40.7
7625
43.4
247008
45.3
108527
47.4
291650
49.6
137975
56.3
211320
56.8
202049
56.9
248127
64.0
287743
65.9
173942
70.9
39388
73.9
240490
0.0
13708
0.9
276964
1.2
229530
1.3
169498
1.5
105442
2.4
28513
4.8
206199
6.5
239040
13.0
207821
17.8
137872
20.7
36767
0.0
282087
0.9
76529
1.5
195260
1.6
226191
2.3
29336
2.6
244852
2.9
296897
177282
3.7
37466
4.0
126376
4.1
150591
10.7
138240
11.2
40351
15.8
108157
20.1
28513
21.2
257253
23.5
32670
30.2
70866
33.1
209611
38.2
167551
40.1
47070
42.7
252713
43.1
87734
45.1
93621
45.5
256816
48.6
286202
48.9
267424
52.8
239040
54.2
94650
56.0
155321
57.8
153612
58.0
283247
59.3
94776
60.5
36914
60.7
161061
62.8
263725
63.0
173942
70.1
16132
71.1
69867
73.0
35278
73.7
125895
0.0
228052
6.2
185055
9.6
10616
15.2
113032
15.5
166845
21.1
271692
31.1
242728
31.8
153129
33.1
178686
34.0
196515
37.4
284391
38.4
120090
39.1
115686
39.8
121886
40.3
273900
44.6
117666
46.5
237030
51.3
132523
55.6
196852
58.2
121743
58.8
53613
60.2
60411
62.0
37118
86.9
G/F12
264816
0.0
101355
5.2
133406
6.6
287358
15.6
255004
18.9
286855
20.8
185055
21.8
258140
33.3
133704
34.5
237498
38.1
205232
44.6
54678
48.0
277728
50.0
254101
50.6
119033
52.5
135190
53.1
126497
54.6
271358
54.7
212874
56.8
300699
58.1
73205
58.6
184295
60.7
36770
61.0
253165
61.3
256780
61.6
237030
62.6
217821
63.1
302968
64.3
103522
66.0
174628
67.6
121743
68.1
251328
70.0
130162
71.3

## Slide 7
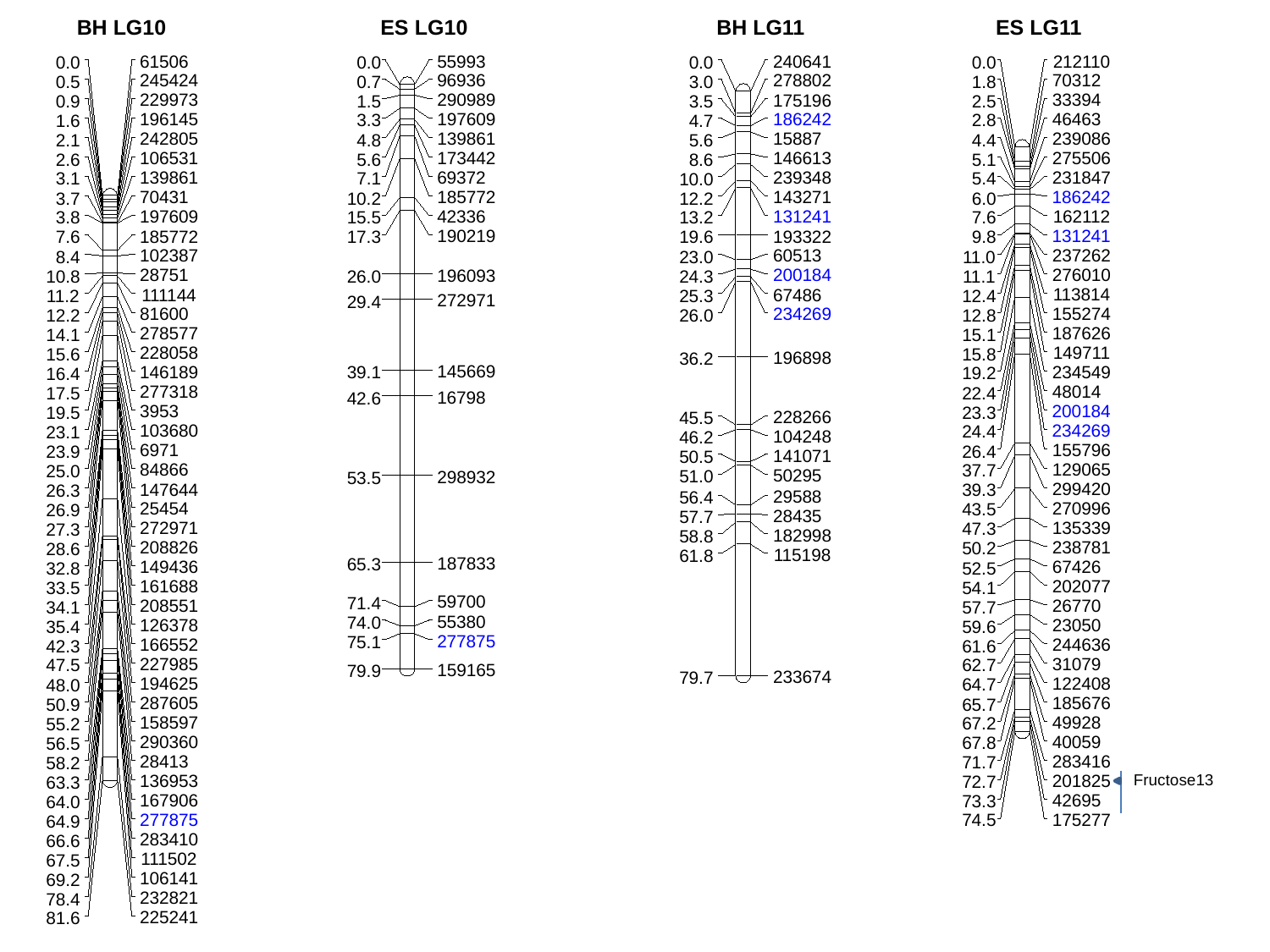

BH LG10
ES LG10
BH LG11
ES LG11
61506
0.0
245424
0.5
229973
0.9
196145
1.6
242805
2.1
106531
2.6
139861
3.1
70431
3.7
197609
3.8
185772
7.6
102387
8.4
28751
10.8
111144
11.2
81600
12.2
278577
14.1
228058
15.6
146189
16.4
277318
17.5
3953
19.5
103680
23.1
6971
23.9
84866
25.0
147644
26.3
25454
26.9
272971
27.3
208826
28.6
149436
32.8
161688
33.5
208551
34.1
126378
35.4
166552
42.3
227985
47.5
194625
48.0
287605
50.9
158597
55.2
290360
56.5
28413
58.2
136953
63.3
167906
64.0
277875
64.9
283410
66.6
111502
67.5
106141
69.2
232821
78.4
225241
81.6
55993
0.0
96936
0.7
290989
1.5
197609
3.3
139861
4.8
173442
5.6
69372
7.1
185772
10.2
42336
15.5
190219
17.3
196093
26.0
272971
29.4
145669
39.1
16798
42.6
298932
53.5
187833
65.3
59700
71.4
55380
74.0
277875
75.1
159165
79.9
240641
0.0
278802
3.0
175196
3.5
186242
4.7
15887
5.6
146613
8.6
239348
10.0
143271
12.2
131241
13.2
193322
19.6
60513
23.0
200184
24.3
67486
25.3
234269
26.0
196898
36.2
228266
45.5
104248
46.2
141071
50.5
50295
51.0
29588
56.4
28435
57.7
182998
58.8
115198
61.8
233674
79.7
212110
0.0
70312
1.8
33394
2.5
46463
2.8
239086
4.4
275506
5.1
231847
5.4
186242
6.0
162112
7.6
131241
9.8
237262
11.0
276010
11.1
113814
12.4
155274
12.8
187626
15.1
149711
15.8
234549
19.2
48014
22.4
200184
23.3
234269
24.4
155796
26.4
129065
37.7
299420
39.3
270996
43.5
135339
47.3
238781
50.2
67426
52.5
202077
54.1
26770
57.7
23050
59.6
244636
61.6
31079
62.7
122408
64.7
185676
65.7
49928
67.2
40059
67.8
283416
71.7
201825
72.7
42695
73.3
175277
74.5
Fructose13

## Slide 8
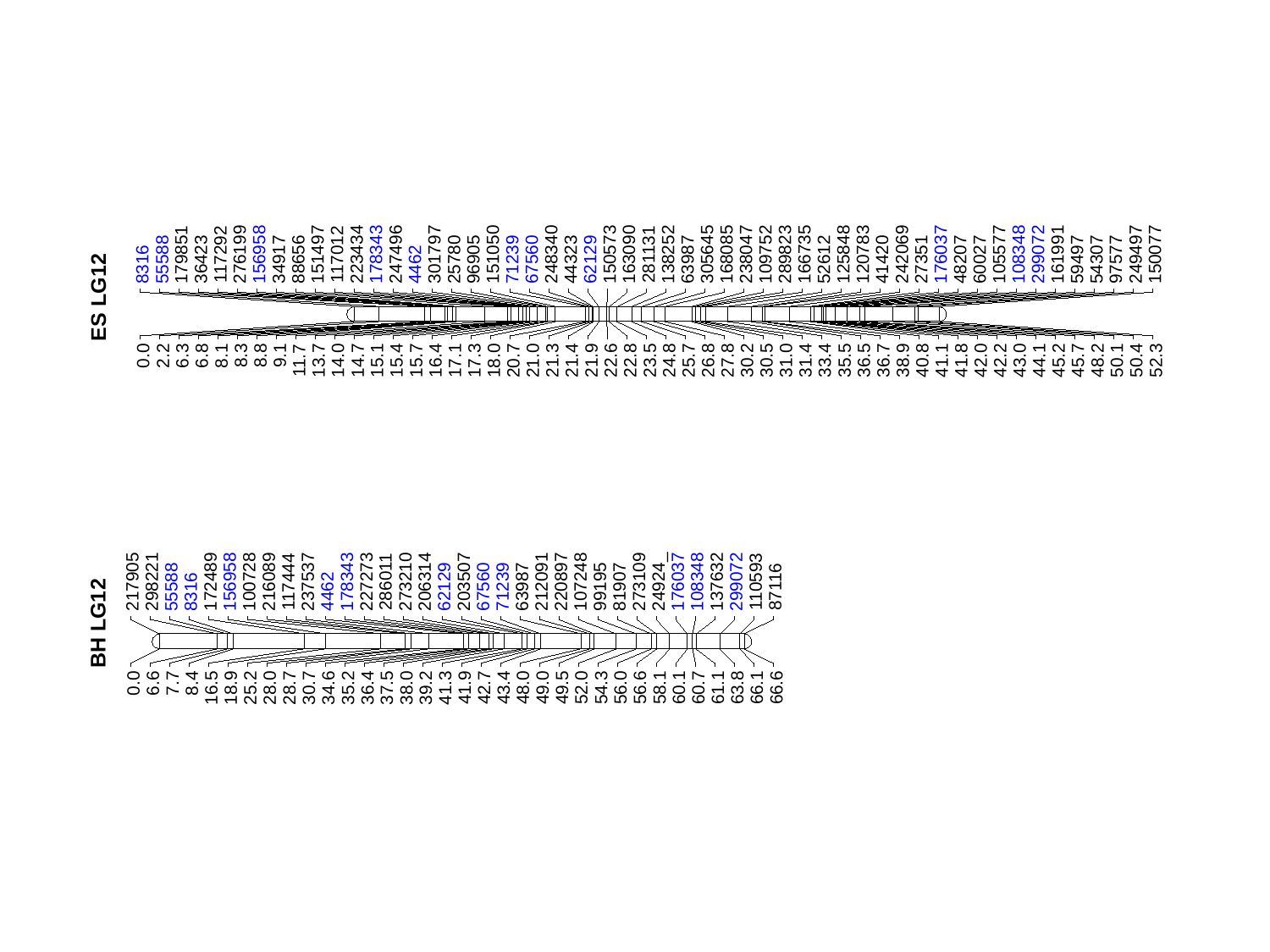

BH LG12
ES LG12
217905
0.0
298221
6.6
55588
7.7
8316
8.4
172489
16.5
156958
18.9
100728
25.2
216089
28.0
117444
28.7
237537
30.7
4462
34.6
178343
35.2
227273
36.4
286011
37.5
273210
38.0
206314
39.2
62129
41.3
203507
41.9
67560
42.7
71239
43.4
63987
48.0
212091
49.0
220897
49.5
107248
52.0
99195
54.3
81907
56.0
273109
56.6
24924_
58.1
176037
60.1
108348
60.7
137632
61.1
299072
63.8
110593
66.1
87116
66.6
8316
0.0
55588
2.2
179851
6.3
36423
6.8
117292
8.1
276199
8.3
156958
8.8
34917
9.1
88656
11.7
151497
13.7
117012
14.0
223434
14.7
178343
15.1
247496
15.4
4462
15.7
301797
16.4
25780
17.1
96905
17.3
151050
18.0
71239
20.7
67560
21.0
248340
21.3
44323
21.4
62129
21.9
150573
22.6
163090
22.8
281131
23.5
138252
24.8
63987
25.7
305645
26.8
168085
27.8
238047
30.2
109752
30.5
289823
31.0
166735
31.4
52612
33.4
125848
35.5
120783
36.5
41420
36.7
242069
38.9
27351
40.8
176037
41.1
48207
41.8
60027
42.0
105577
42.2
108348
43.0
299072
44.1
161991
45.2
59497
45.7
54307
48.2
97577
50.1
249497
50.4
150077
52.3

## Slide 9
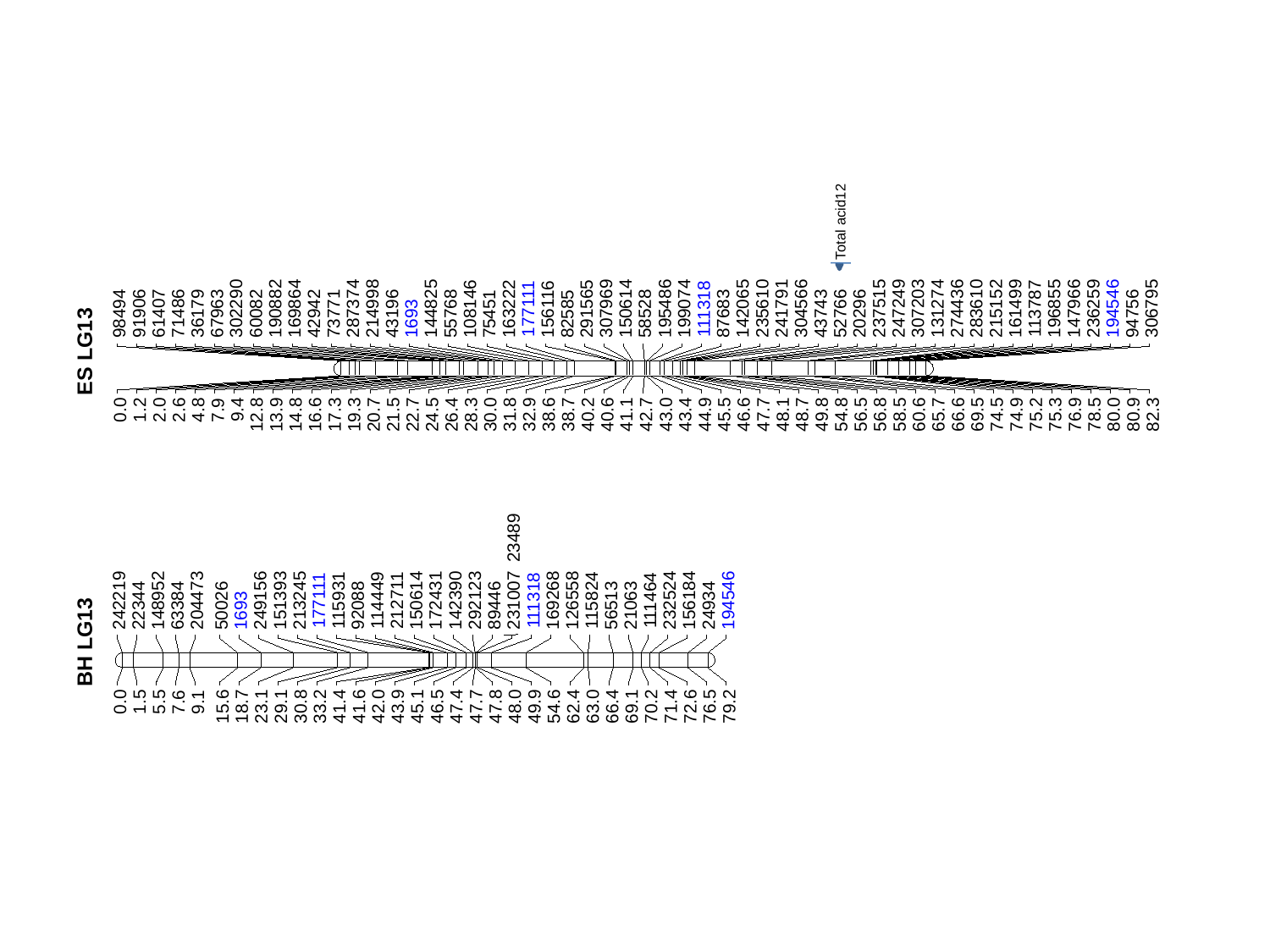

98494
0.0
91906
1.2
61407
2.0
71486
2.6
36179
4.8
67963
7.9
302290
9.4
60082
12.8
190882
13.9
169864
14.8
42942
16.6
73771
17.3
287374
19.3
214998
20.7
43196
21.5
1693
22.7
144825
24.5
55768
26.4
108146
28.3
75451
30.0
163222
31.8
177111
32.9
156116
38.6
82585
38.7
291565
40.2
307969
40.6
150614
41.1
58528
42.7
195486
43.0
199074
43.4
111318
44.9
87683
45.5
142065
46.6
235610
47.7
241791
48.1
304566
48.7
43743
49.8
52766
54.8
20296
56.5
237515
56.8
247249
58.5
307203
60.6
131274
65.7
274436
66.6
283610
69.5
215152
74.5
161499
74.9
113787
75.2
196855
75.3
147966
76.9
236259
78.5
194546
80.0
94756
80.9
306795
82.3
Total acid12
242219
0.0
22344
1.5
148952
5.5
63384
7.6
204473
9.1
50026
15.6
1693
18.7
249156
23.1
151393
29.1
213245
30.8
177111
33.2
115931
41.4
92088
41.6
114449
42.0
212711
43.9
150614
45.1
172431
46.5
142390
47.4
292123
47.7
89446
47.8
231007
23489
48.0
111318
49.9
169268
54.6
126558
62.4
115824
63.0
56513
66.4
21063
69.1
111464
70.2
232524
71.4
156184
72.6
24934
76.5
194546
79.2
ES LG13
BH LG13

## Slide 10
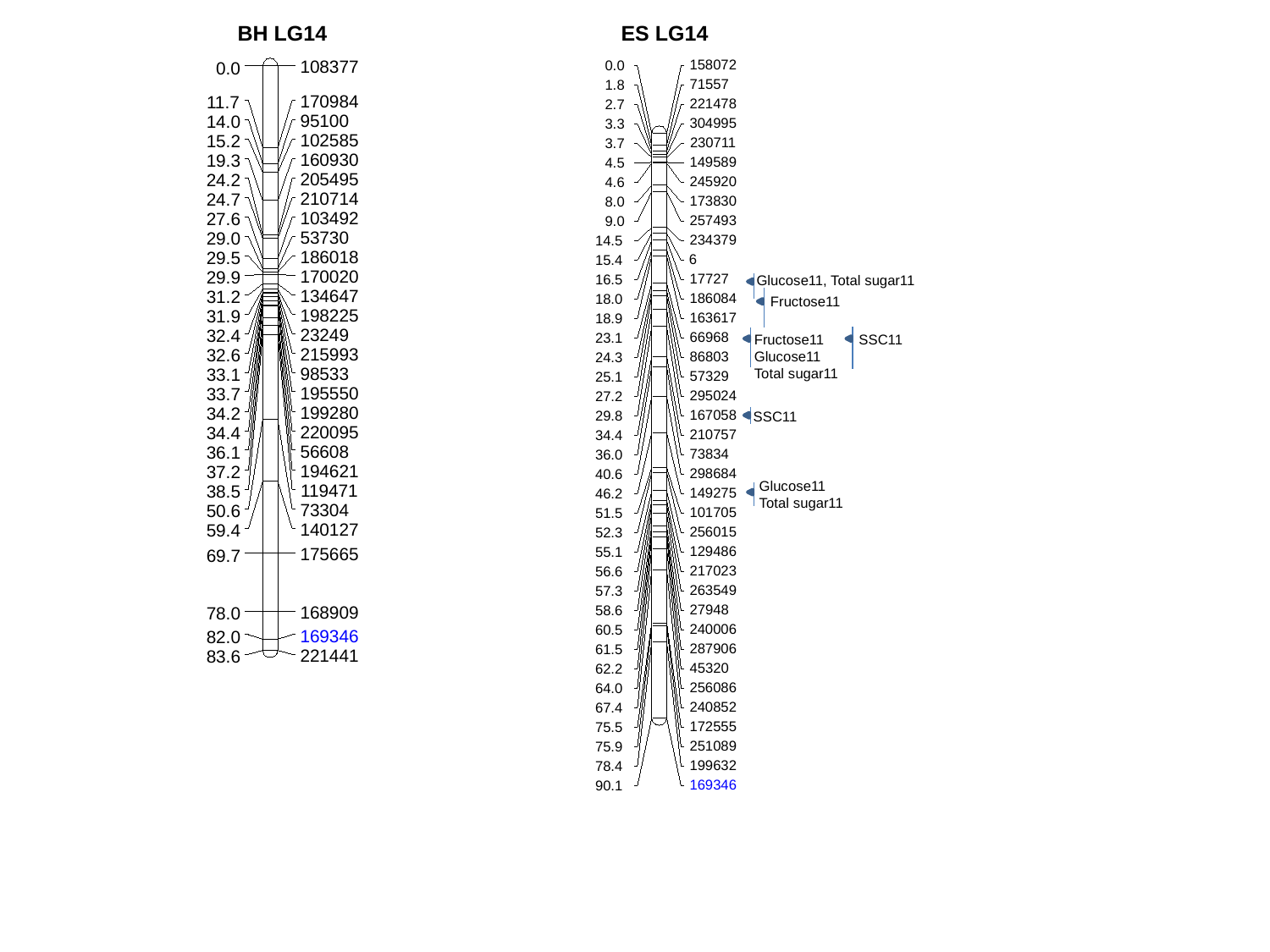

BH LG14
ES LG14
108377
0.0
170984
11.7
95100
14.0
102585
15.2
160930
19.3
205495
24.2
210714
24.7
103492
27.6
53730
29.0
186018
29.5
170020
29.9
134647
31.2
198225
31.9
23249
32.4
215993
32.6
98533
33.1
195550
33.7
199280
34.2
220095
34.4
56608
36.1
194621
37.2
119471
38.5
73304
50.6
140127
59.4
175665
69.7
168909
78.0
169346
82.0
221441
83.6
158072
0.0
71557
1.8
221478
2.7
304995
3.3
230711
3.7
149589
4.5
245920
4.6
173830
8.0
257493
9.0
234379
14.5
6
15.4
17727
16.5
186084
18.0
163617
18.9
66968
23.1
86803
24.3
57329
25.1
295024
27.2
167058
29.8
210757
34.4
73834
36.0
298684
40.6
149275
46.2
101705
51.5
256015
52.3
129486
55.1
217023
56.6
263549
57.3
27948
58.6
240006
60.5
287906
61.5
45320
62.2
256086
64.0
240852
67.4
172555
75.5
251089
75.9
199632
78.4
169346
90.1
Fructose11
Glucose11, Total sugar11
Fructose11
Glucose11
Total sugar11
SSC11
SSC11
Glucose11
Total sugar11

## Slide 11
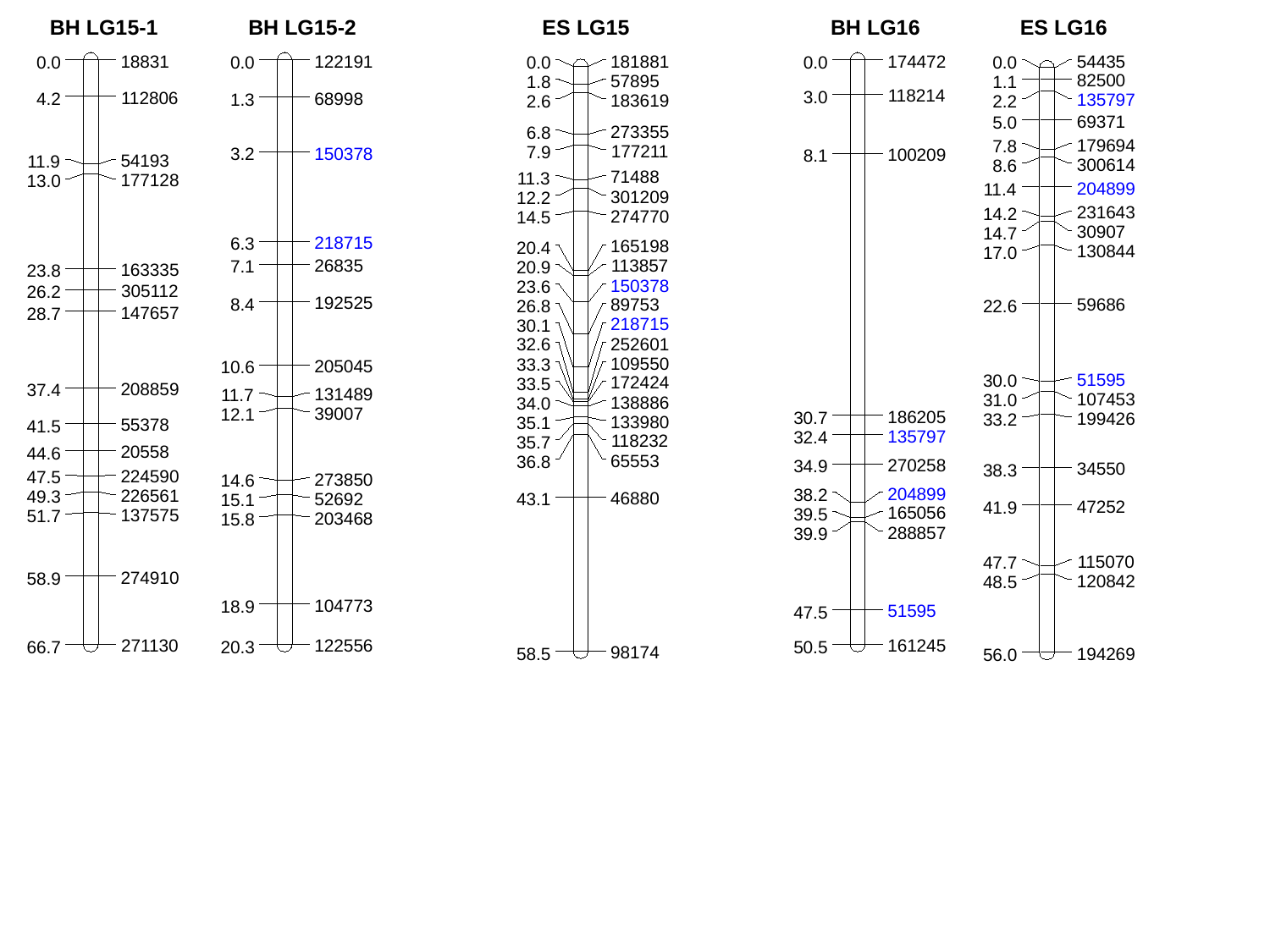

BH LG15-1
BH LG15-2
ES LG15
BH LG16
ES LG16
18831
0.0
112806
4.2
54193
11.9
177128
13.0
163335
23.8
305112
26.2
147657
28.7
208859
37.4
55378
41.5
20558
44.6
224590
47.5
226561
49.3
137575
51.7
274910
58.9
271130
66.7
122191
0.0
68998
1.3
150378
3.2
218715
6.3
26835
7.1
192525
8.4
205045
10.6
131489
11.7
39007
12.1
273850
14.6
52692
15.1
203468
15.8
104773
18.9
122556
20.3
181881
0.0
57895
1.8
183619
2.6
273355
6.8
177211
7.9
71488
11.3
301209
12.2
274770
14.5
165198
20.4
113857
20.9
150378
23.6
89753
26.8
218715
30.1
252601
32.6
109550
33.3
172424
33.5
138886
34.0
133980
35.1
118232
35.7
65553
36.8
46880
43.1
98174
58.5
174472
0.0
118214
3.0
100209
8.1
186205
30.7
135797
32.4
270258
34.9
204899
38.2
165056
39.5
288857
39.9
51595
47.5
161245
50.5
54435
0.0
82500
1.1
135797
2.2
69371
5.0
179694
7.8
300614
8.6
204899
11.4
231643
14.2
30907
14.7
130844
17.0
59686
22.6
51595
30.0
107453
31.0
199426
33.2
34550
38.3
47252
41.9
115070
47.7
120842
48.5
194269
56.0

## Slide 12
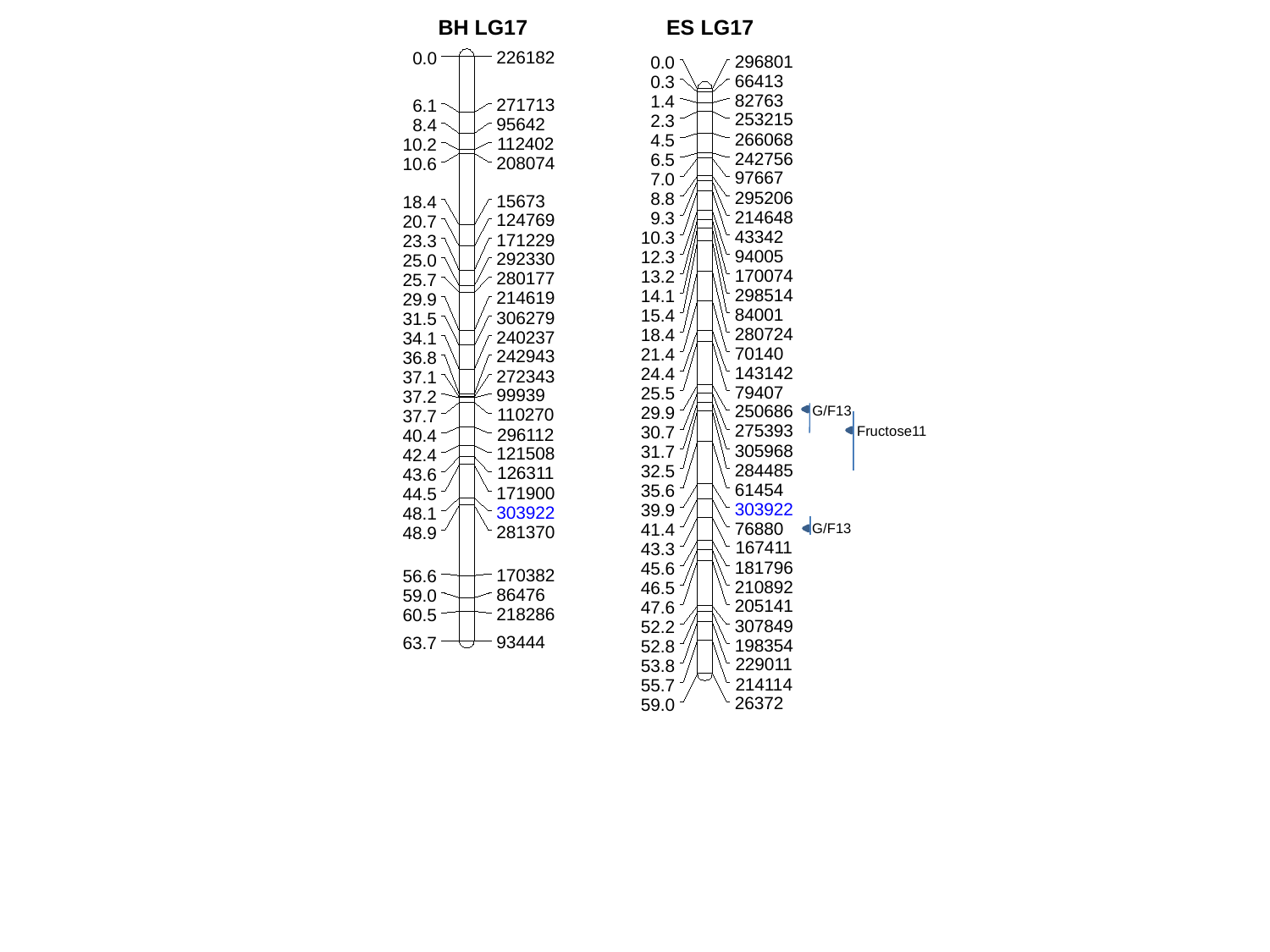

BH LG17
ES LG17
226182
0.0
271713
6.1
95642
8.4
112402
10.2
208074
10.6
15673
18.4
124769
20.7
171229
23.3
292330
25.0
280177
25.7
214619
29.9
306279
31.5
240237
34.1
242943
36.8
272343
37.1
99939
37.2
110270
37.7
296112
40.4
121508
42.4
126311
43.6
171900
44.5
303922
48.1
281370
48.9
170382
56.6
86476
59.0
218286
60.5
93444
63.7
296801
0.0
66413
0.3
82763
1.4
253215
2.3
266068
4.5
242756
6.5
97667
7.0
295206
8.8
214648
9.3
43342
10.3
94005
12.3
170074
13.2
298514
14.1
84001
15.4
280724
18.4
70140
21.4
143142
24.4
79407
25.5
250686
29.9
275393
30.7
305968
31.7
284485
32.5
61454
35.6
303922
39.9
76880
41.4
167411
43.3
181796
45.6
210892
46.5
205141
47.6
307849
52.2
198354
52.8
229011
53.8
214114
55.7
26372
59.0
G/F13
Fructose11
G/F13

## Slide 13
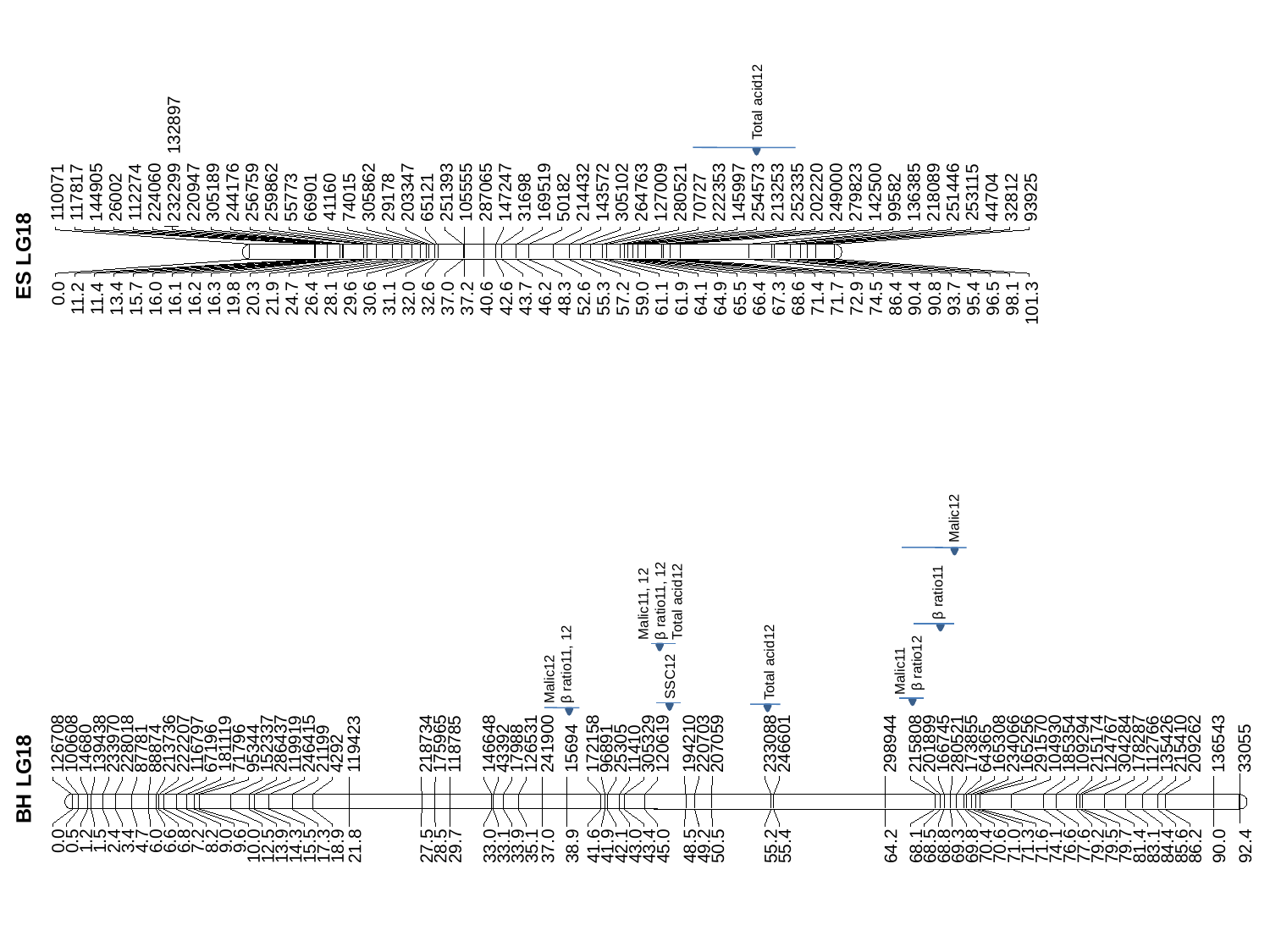

110071
0.0
117817
11.2
144905
11.4
26002
13.4
112274
15.7
224060
16.0
232299
132897
16.1
220947
16.2
305189
16.3
244176
19.8
256759
20.3
259862
21.9
55773
24.7
66901
26.4
41160
28.1
74015
29.6
305862
30.6
29178
31.1
203347
32.0
65121
32.6
251393
37.0
105555
37.2
287065
40.6
147247
42.6
31698
43.7
169519
46.2
50182
48.3
214432
52.6
143572
55.3
305102
57.2
264763
59.0
127009
61.1
280521
61.9
70727
64.1
222353
64.9
145997
65.5
254573
66.4
213253
67.3
252335
68.6
202220
71.4
249000
71.7
279823
72.9
142500
74.5
99582
86.4
136385
90.4
218089
90.8
251446
93.7
253115
95.4
44704
96.5
32812
98.1
93925
101.3
Total acid12
126708
0.0
100608
0.5
14680
1.2
139438
1.5
233970
2.4
228018
3.4
87781
4.7
88874
6.0
213736
6.6
222207
6.8
116797
7.2
67196
8.2
181119
9.0
71796
9.6
95344
10.0
152337
12.5
286437
13.9
119919
14.3
246415
15.5
21199
17.3
4292
18.9
119423
21.8
218734
27.5
175965
28.5
118785
29.7
146648
33.0
43392
33.1
17988
33.9
126531
35.1
241900
37.0
15694
38.9
172158
41.6
96891
41.9
25305
42.1
11410
43.0
305329
43.4
120619
45.0
194210
48.5
220703
49.2
207059
50.5
233088
55.2
246601
55.4
298944
64.2
215808
68.1
201899
68.5
166745
68.8
280521
69.3
173855
69.8
64365
70.4
165308
70.6
234066
71.0
165256
71.3
291570
71.6
104930
74.1
185354
76.6
109294
77.6
215174
79.2
124767
79.5
304284
79.7
178287
81.4
112766
83.1
135426
84.4
215410
85.6
209262
86.2
136543
90.0
33055
92.4
ES LG18
Malic12
Malic11, 12
β ratio11, 12
Total acid12
β ratio11
Total acid12
Malic11
 β ratio12
SSC12
Malic12
β ratio11, 12
BH LG18

## Slide 14
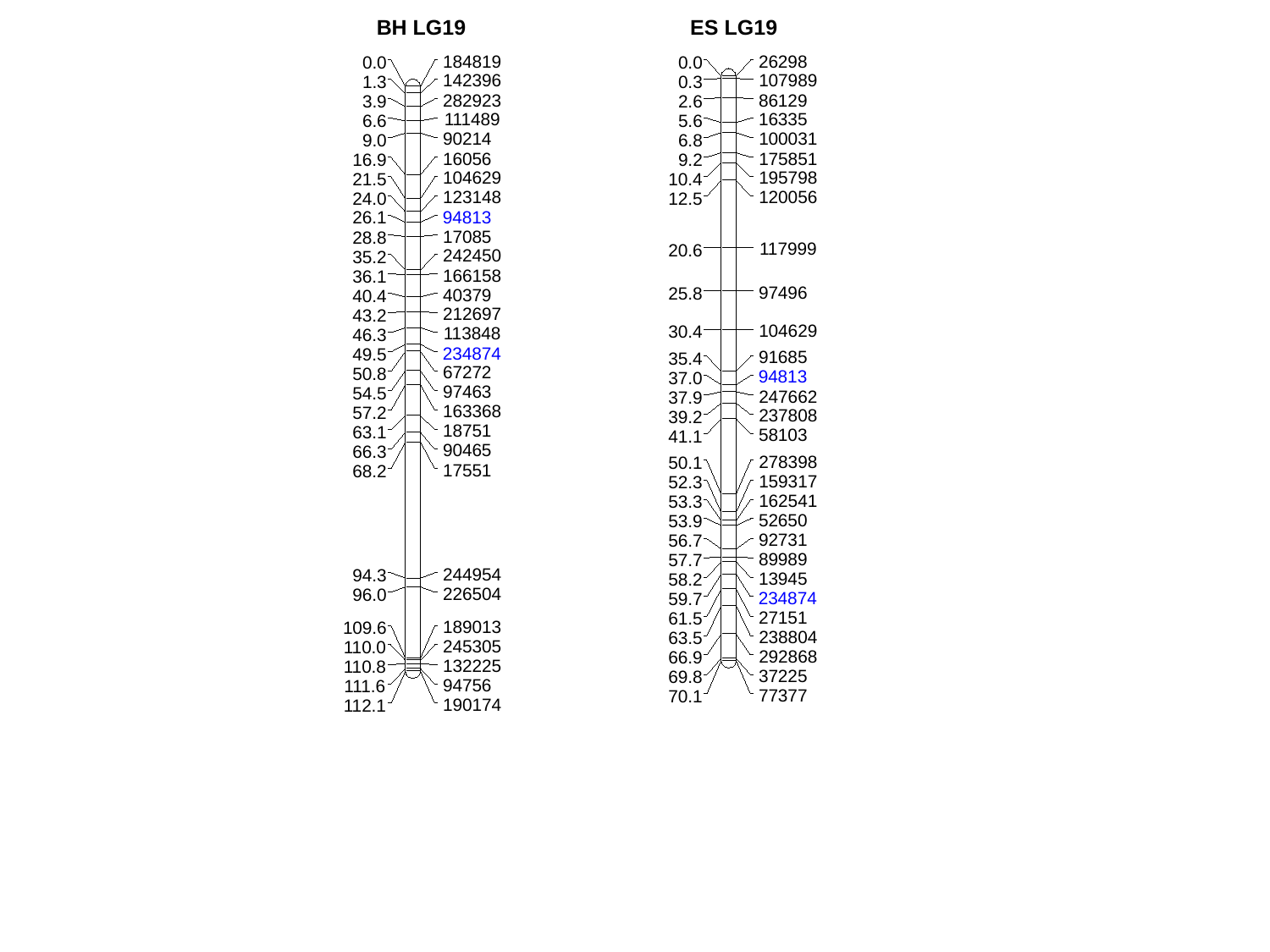

BH LG19
ES LG19
184819
0.0
142396
1.3
282923
3.9
111489
6.6
90214
9.0
16056
16.9
104629
21.5
123148
24.0
94813
26.1
17085
28.8
242450
35.2
166158
36.1
40379
40.4
212697
43.2
113848
46.3
234874
49.5
67272
50.8
97463
54.5
163368
57.2
18751
63.1
90465
66.3
17551
68.2
244954
94.3
226504
96.0
189013
109.6
245305
110.0
132225
110.8
94756
111.6
190174
112.1
26298
0.0
107989
0.3
86129
2.6
16335
5.6
100031
6.8
175851
9.2
195798
10.4
120056
12.5
117999
20.6
97496
25.8
104629
30.4
91685
35.4
94813
37.0
247662
37.9
237808
39.2
58103
41.1
278398
50.1
159317
52.3
162541
53.3
52650
53.9
92731
56.7
89989
57.7
13945
58.2
234874
59.7
27151
61.5
238804
63.5
292868
66.9
37225
69.8
77377
70.1
