# Supplementary material for: Construction of a high-density genetic map and QTLs mapping for sugars and acids in grape berries
Source: BMC Plant Biol. 2015 Feb 3;15:28. doi: 10.1186/s12870-015-0428-2 (PMC4329212; doi:10.1186/s12870-015-0428-2)
Supplement: Additional file 4: Figure S3. — Positions of SNP-based markers on genetic maps, and their physical positions on the reference genome. [file 12870_2015_428_MOESM4_ESM.docx]

**Figure S3.** **Positions of SNP-based markers on genetic maps, and their physical positions on the reference genome.** The maternal parent is ‘Beihong’ (BH, blue triangles) and the paternal parent ‘E.S.7-11-49’ (ES, black squares).
